# STING regulates metabolic reprogramming in macrophages via HIF-1α during *Brucella* infection

**DOI:** 10.1371/journal.ppat.1009597

**Published:** 2021-05-14

**Authors:** Marco Tulio R. Gomes, Erika S. Guimarães, Fabio V. Marinho, Isabella Macedo, Eric R. G. R. Aguiar, Glen N. Barber, Pedro M. M. Moraes-Vieira, José Carlos Alves-Filho, Sergio C. Oliveira

**Affiliations:** 1 Departamento de Bioquímica e Imunologia, Instituto de Ciências Biológicas, Universidade Federal de Minas Gerais, Belo Horizonte, Minas Gerais, Brazil; 2 Departamento de Genética, Ecologia e Evolução, Programa de Pós-Graduação em Genética, Instituto de Ciências Biológicas, Universidade Federal de Minas Gerais, Belo Horizonte, Minas Gerais, Brazil; 3 Departmento de Ciências Biológicas, Centro de Biotecnologia e Genética, Universidade Estadual de Santa Cruz, Ilhéus, Bahia, Brazil; 4 Department of Cell Biology, University of Miami, Miami, Florida; 5 Departmento de Genética, Evolução, Microbiologia e Imunologia, Universidade Estadual de Campinas, Campinas São Paulo, Brazil; 6 Departmento de Farmacologia, Faculdade de Medicina de Ribeirão Preto, Universidade de São Paulo, Ribeirão Preto, São Paulo, Brazil; 7 Instituto Nacional de Ciência e Tecnologia em Doenças Tropicais (INCT-DT), CNPq MCT, Salvador, Bahia, Brazil; University of California, Davis, UNITED STATES

## Abstract

Macrophages metabolic reprogramming in response to microbial insults is a major determinant of pathogen growth or containment. Here, we reveal a distinct mechanism by which stimulator of interferon genes (STING), a cytosolic sensor that regulates innate immune responses, contributes to an inflammatory M1-like macrophage profile upon *Brucella abortus* infection. This metabolic reprogramming is induced by STING-dependent stabilization of hypoxia-inducible factor-1 alpha (HIF-1α), a global regulator of cellular metabolism and innate immune cell functions. HIF-1α stabilization reduces oxidative phosphorylation and increases glycolysis during infection with *B*. *abortus* and, likewise, enhances nitric oxide production, inflammasome activation and IL-1β release in infected macrophages. Furthermore, the induction of this inflammatory profile participates in the control of bacterial replication since absence of HIF-1α renders mice more susceptible to *B*. *abortus* infection. Mechanistically, activation of STING by *B*. *abortus* infection drives the production of mitochondrial reactive oxygen species (mROS) that ultimately influences HIF-1α stabilization. Moreover, STING increases the intracellular succinate concentration in infected macrophages, and succinate pretreatment induces HIF-1α stabilization and IL-1β release independently of its cognate receptor GPR91. Collectively, these data demonstrate a pivotal mechanism in the immunometabolic regulation of macrophages during *B*. *abortus* infection that is orchestrated by STING via HIF-1α pathway and highlight the metabolic reprogramming of macrophages as a potential treatment strategy for bacterial infections.

## Author summary

The impact of host cell metabolism on pathogen growth or restriction represent an emerging field in immunology and shed light on the intricate network of signaling pathways during immune cells response. Here, we dissected a distinct mechanism by which STING regulates macrophage metabolic reprogramming eliciting an inflammatory profile during *Brucella* infection. *Brucella abortus* is an intracellular bacterium that causes brucellosis, an infectious disease that promotes abortion in domestic animals leading to severe economic losses and an inflammatory condition in humans. The metabolite reprogramming orchestrated by STING relies on HIF-1α stabilization through increased succinate and mROS levels. We demonstrated that HIF-1α stabilization enhances nitric oxide production, inflammasome activation and IL-1β release in infected macrophages, and this inflammatory profile participates in the control of bacterial replication. Thus, our findings bring new insights on this intricate circuit by which the host immune senses intracellular pathogens contributing to development of drugs and/or vaccines to control infectious diseases.

## Introduction

Over the past decade, immunometabolism, or how changes in metabolic processes regulate immune cell responses, gained appreciation and is now recognized as a rising field in immunology [1]. Of particular interest is the metabolic reprogramming of macrophages, i.e. the key metabolic and functional differences between the so-called M1- and M2-like macrophages, a polarized simplified model for a reality of much greater dynamism and complexity, commonly associated with an inflammatory or anti-inflammatory status, respectively [2]. In this regard, the M1-like macrophage profile is characterized by the production of inflammatory cytokines, such as IL-1β, by nitric oxide (NO) production through inducible nitric oxide synthase (NOS2), and by efficient generation of reactive oxygen species (ROS) [2]. Furthermore, energy production in M1-like macrophages shifts from mitochondrial oxidative phosphorylation (OXPHOS) to a marked upregulation of aerobic glycolytic metabolism (a process well-known as the Warburg effect) to support phagocytic and microbicidal function [3].

The impaired tricarboxylic acid (TCA) cycle in M1-like macrophages drives the accumulation of several metabolites, including succinate, a metabolite recognized for its role in innate immune signaling [4,5]. Succinate accumulation perturbs immune function by stabilizing hypoxia-inducible factor 1α (HIF-1α) [6–8], a transcription factor subunit that forms a heterodimer with the stable HIF-1β [9]. Mechanistically, HIF-1α stability is controlled by the hydroxylation of proline residues promoted by prolyl hydroxylase (PHD). Once the proline residues on the HIF-1α subunit are hydroxylated, the protein is marked for ubiquitin-proteasome mediated degradation [10]. Succinate excess impairs PHD activity leading to HIF-1α stabilization and activation, which facilitates the metabolic shift from OXPHOS to glycolysis, and also sustains the inflammatory phenotype by inducing the production of inflammatory cytokines such as IL-1β [6,10]. In addition, accumulated succinate can be oxidized to fumarate by succinate dehydrogenase (SDH) driving mitochondrial ROS (mROS) production through reverse electron transport (RET) from complex II to complex I, leading to mROS-dependent HIF-1α stabilization [11]. Moreover, extracellular succinate can also activate its cognate receptor SUCNR1/GPR91 (G protein–coupled receptor 91) that acts in synergy with TLR ligands, driving inflammatory cytokine production [12]. Due to this set of features, M1-like macrophages are considered as inflammatory and display enhanced antimicrobial activity [2]. In contrast, M2-like macrophages are associated with the generation of anti-inflammatory products such as glucocorticoids, IL-10 and IL-13 and show an intact TCA cycle and enhanced OXPHOS activity [2]. The M2-like profile is more associated with tissue remodeling and wound healing and is considered in particular cases as anti-inflammatory [13,14].

*Brucella abortus* is a Gram-negative bacterium that causes brucellosis, a worldwide zoonotic disease. In humans, the disease is characterized by undulant fever, and chronic debilitating symptoms such as arthritis, endocarditis, and meningitis; in cattle, it causes abortion and infertility [15,16]. Although brucellosis is known for almost 200 years, the disease is still difficult to diagnose and very challenging to treat, requiring prolonged courses of multiple antibiotics, which culminates in a high rate of relapse and a major public health burden [17,18]. A better understanding of the host-pathogen interplay that enables *Brucella* persistence is crucial for the development of an effective treatment for brucellosis. The metabolic divergence of different macrophage phenotypes is of particular relevance to the study of intracellular infections since it can directly affect pathogen survival and is likely to be a key factor in determining disease control or progression [2,19,20]. Indeed, human-like macrophages infected with *Brucella* undergo a metabolic shift that resembles the M1-like macrophage Warburg effect [21]. However, *Brucella* survives and replicates preferentially in M2-like macrophages due to the increased availability of glucose within this niche [20]. Thus, an immunometabolic approach will shed light on the activation of immune pathways during *Brucella* infection in view of a potential therapy for restricting bacterial proliferation and preventing chronic infection.

STING is a transmembrane protein that regulates DNA-mediated type I interferon-dependent innate immune responses [22]. During intracellular bacterial infection, STING can function as a direct sensor of bacterial cyclic dinucleotides (CDNs) as well as an adaptor molecule in DNA recognition [23]. STING is important for the control of *B*. *abortus* infection, since STING knockout (KO) mice are more susceptible to the infection when compared to wild-type (WT) animals [24]. Furthermore, we have reported that STING plays a central role during *B*. *abortus* infection by directly detecting bacterial CDNs and consequentially triggering an inflammatory response that includes regulation of NOS2 expression [25]. Therefore, we aimed to determine STING involvement in regulating macrophage metabolic reprogramming during *B*. *abortus* infection. Here, we demonstrated that STING participates in the induction of the M1-like macrophage phenotype in infected cells, a process that is dependent on HIF-1α stabilization through increased succinate and mROS levels.

## Results

### STING contributes to the inflammatory profile in macrophages

**Fig 1:**
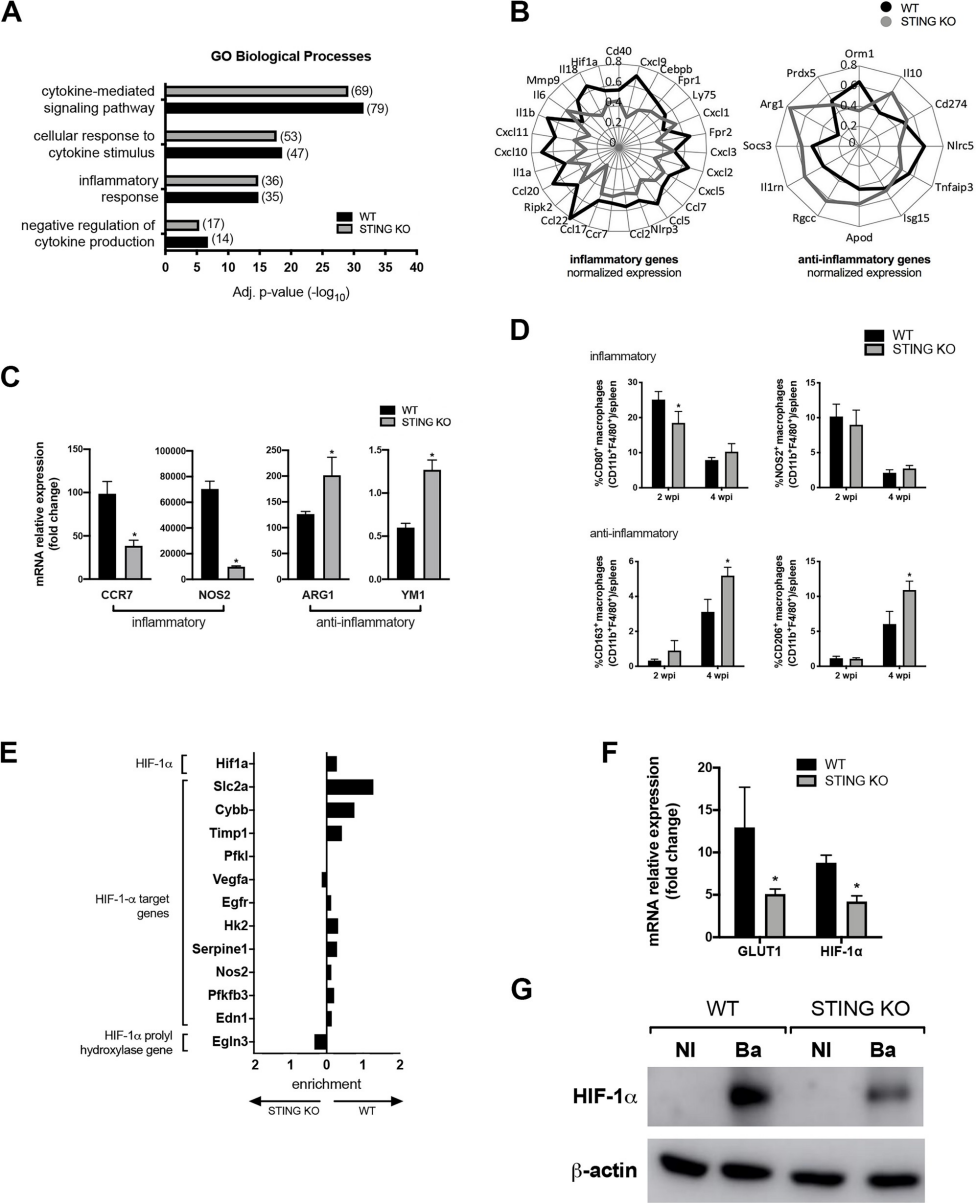
STING contributes to an inflammatory profile in macrophages infected with *B*. *abortus*. **(A)** Gene ontology enrichment analysis of differentially expressed genes from infected C57BL/6 (WT) or STING KO macrophages. The number of genes related to enriched GO biological process is indicated in each case. **(B)** Fold change of inflammatory and anti-inflammatory genes upregulated in infected C57BL/6 (WT) or STING KO macrophages. The numbers in radial axes represent the value obtained after the normalization between genotypes of the fold change observed for each gene. **(C)** CCR7, NOS2, ARG1, and YM1 expression levels determined by real-time RT-PCR in *B*. *abortus*-infected macrophages derived from C57BL/6 (WT) or STING KO mice. **(D)** Frequency of CD80^+^F4/80^+^CD11b^+^, NOS2^+^F4/80^+^CD11b^+^, CD163^+^F4/80^+^CD11b^+^ and CD206^+^F4/80^+^CD11b^+^ cells measured by flow cytometry analysis in spleen cells from infected C57BL/6 (WT) and STING KO mice, at 2 or 4 weeks post-infection (wpi). Representative plots of analysis are shown in S3 Fig. **(E)** Fold change of genes from infected C57BL/6 (WT) or STING KO macrophages represented in the enriched HIF-1α pathway assigned by KEGG database analysis. Gene fold change is represented normalized between groups. **(F)** GLUT1 and HIF-1α expression levels determined by real-time RT-PCR in *B*. *abortus*-infected macrophages derived from C57BL/6 (WT) or STING KO mice. **(G)** Western blot analysis of HIF-1α in cell lysates from macrophages derived from C57BL/6 (WT) or STING KO mice, non-infected (NI) or infected with *B*. *abortus* (Ba). Equal loading was controlled by measuring β-actin in the corresponding cell lysates. The data (C-D and F-G) are representative of three independent experiments. The data (C-D and F) are presented as mean ± SD, *p < 0.05, Student’s t test.

The macrophage metabolic profile that emerges during *B*. *abortus* replication is critical for the outcome of infection, allowing its persistence or growth restriction in the host environment [20]. STING is an adaptor molecule crucial to trigger an appropriate immune response to control *B*. *abortus* infection [25]. Therefore, we decided to investigate whether STING signaling during bacterial infection regulates the macrophage metabolic profile which is commonly associated with an inflammatory or anti-inflammatory status [2]. For that, we used the data from a microarray analysis performed earlier by our group, in which the upregulated and downregulated genes in *B*. *abortus*-infected macrophages from WT and STING KO mice were determined [25]. Hence, these genes were submitted to biological pathway enrichment using the GO and KEGG databases. Interestingly, this analysis demonstrated that common pathways involved in immune and inflammatory responses against *B*. *abortus* infection were found to be enriched in both WT and STING KO macrophages (Fig 1A). To further explore this data, analysis of fold change of inflammatory and anti-inflammatory genes derived from these enriched pathways were performed, comparing infected WT macrophages with infected STING KO cells. Of particular relevance was the fact that many genes involved with the inflammatory profile were upregulated in WT infected macrophages in comparison with STING KO infected cells. For instance, the genes encoding chemokines and inflammasome-related cytokines *IL-1β* and *IL-18* (Fig 1B). In contrast, when anti-inflammatory genes were analyzed an opposite effect was observed, for instance *IL-10* and M2-marker *ARG1* (arginase-1) (Fig 1B). These data suggest that STING is involved with the full induction of the inflammatory profile. To confirm these findings, we performed quantitative real-time RT-PCR analysis and, in accordance with the microarray data, inflammatory macrophage-related markers, such as CCR7 (C-C chemokine receptor type 7) and NOS2 were reduced in STING KO infected macrophages, while anti-inflammatory macrophage-related markers such as ARG1 and YM1 (chitinase-like 3) were enhanced in STING KO infected macrophages in comparison with WT infected cells (Fig 1C). To determine whether this macrophage polarization was specific for *Brucella* infection, macrophages derived from WT and STING KO mice were treated with cyclic guanosine monophosphate-adenosine monophosphate (cGAMP), a STING activator. The quantitative real-time RT-PCR analysis showed that NOS2 expression level (S1A Fig) was reduced in STING KO stimulated macrophages, while ARG1 expression level (S1B Fig) was unaltered in comparison with WT stimulated cells. These data suggest that activation of STING alone is not sufficient to fully promote the polarization status of macrophages, which indicates that the engagement of other pathways triggered by *Brucella* infection are also involved in the induction of this profile.

Furthermore, we carried out a time course *B*. *abortus* infection experiment to evaluate by flow cytometry the role of STING in macrophage phenotype (inflammatory, or anti-inflammatory) in spleens using different markers of polarization. Gating strategy is shown in S2 Fig and representative 2D-plots of each analysis are shown in S3 Fig. The percentage of CD80^+^ macrophages was reduced in splenic cells from STING KO infected mice at 2 weeks post-infection, albeit no difference was observed concerning NOS2^+^ macrophages (Fig 1D). However, no difference was observed in STING KO versus WT mice at 4 weeks post-infection in the percentage of macrophage inflammatory markers. In contrast, STING KO infected mice displayed an enhanced frequency of anti-inflammatory CD163^+^ and CD206^+^ macrophages at 4 weeks post-infection in comparison with splenic macrophages from WT infected animals (Fig 1D). Altogether, these results demonstrate that STING is affecting the frequency of anti-inflammatory macrophages at later phase of *B*. *abortus* infection and promoting a slight tendency towards the macrophage inflammatory profile during the initial phase of infection.

Such alteration in macrophage function is regularly accompanied by metabolic changes [26]. In that context, HIF-1α arose as an important component of the cellular machinery involved in the control of immune cell behavior and cellular metabolism [27–29]. Therefore, we hypothesized that HIF-1α might be linked to the STING-dependent induction of the inflammatory profile in macrophages. Indeed, function analysis of gene expression in the KEGG database demonstrated that the HIF-1α pathway is enriched in macrophages infected with *B*. *abortus* when compared with non-infected cells (adjusted *p*-value of 1.6e^-6^ for WT and 1.4e^-7^ for STING KO). Additionally, we explored the genes assigned by KEGG analysis that were associated with the HIF-1α pathway. We observed that the majority of HIF-1α target genes and HIF-1α itself were upregulated during *B*. *abortus* infection in WT macrophages when compared with STING KO cells (Fig 1E). Interestingly, the opposite effect was observed regarding the *prolyl hydroxylase* gene which is involved with HIF-1α degradation (Fig 1E). Hence, to investigate the interplay between STING and HIF-1α pathways, we evaluated HIF-1α and GLUT1 (glucose transporter 1) expression, that is regulated by HIF-1α and it is considered a well-known marker for the HIF-1α-induced glycolysis [30,31]. Infected macrophages from STING KO mice showed reduced gene expression of *HIF-1α* and *GLUT1* (Fig 1F). Similarly, *B*. *abortus* induced higher levels of HIF-1α protein expression in WT macrophages compared to STING KO cells (Fig 1G). In addition, non-infected macrophages (WT and KO) showed no detected levels of HIF-1α (Fig 1G). Together these findings demonstrate that STING contributes to establish an inflammatory profile in macrophages during *B*. *abortus* infection and to drive a consistent expression and stabilization of HIF-1α.

### HIF-1α pathway drives metabolic reprogramming in infected macrophages

**Fig 2:**
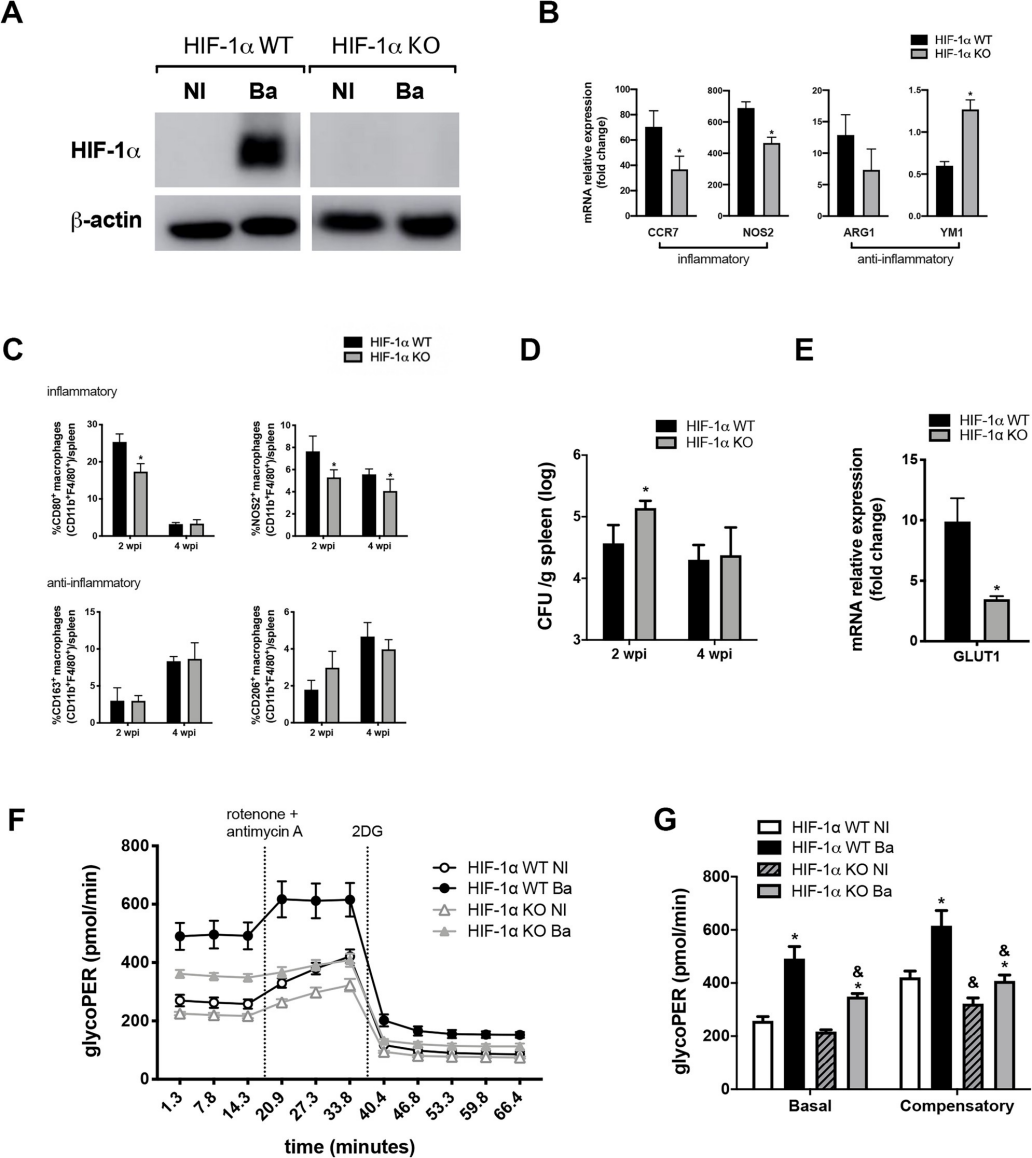
Metabolic reprogramming in infected macrophages requires HIF-1α. **(A)** Western blot analysis of HIF-1α in cell lysates from macrophages derived from HIF-1α WT or HIF-1α KO mice, non-infected (NI) or infected with *B*. *abortus* (Ba). Equal loading was controlled by measuring β-actin in the corresponding cell lysates. **(B)** CCR7, NOS2, ARG1, and YM1 expression levels determined by real-time RT-PCR in *B*. *abortus*-infected macrophages derived from HIF-1α WT or HIF-1α KO mice. **(C)** Frequency of CD80^+^F4/80^+^CD11b^+^, NOS2^+^F4/80^+^CD11b^+^, CD163^+^F4/80^+^CD11b^+^ and CD206^+^F4/80^+^CD11b^+^ cells measured by flow cytometry analysis in spleen cells from infected HIF-1α WT or HIF-1α KO mice, at 2 or 4 weeks post-infection (wpi). Representative plots of analysis are shown in S4 Fig. **(D)** CFU numbers in spleen homogenates of HIF-1α WT and HIF-1α KO mice infected intraperitoneally with *B*. *abortus* at 2 or 4 weeks post-infection (wpi). **(E)** GLUT1 expression levels determined by real-time RT-PCR in *B*. *abortus* infected-macrophages derived from HIF-1α WT and HIF-1α KO mice. **(F)** Time-course quantification of glycolytic proton efflux rate (glycoPER) in macrophages derived from HIF-1α WT and HIF-1α KO mice, non-infected (NI) or infected with *B*. *abortus* (Ba). **(G)** Quantification of basal and compensatory glycoPER. The data are representative of three (A-E) or two (F and G) independent experiments. The data (B-C and E) are presented as mean ± SD, *p < 0.05, Student’s t test. The data (D) is presented as mean ± SD, *p < 0.05, two-way ANOVA. The data (G) is presented as mean ± SD, * (comparison between NI and Ba) or & (comparison between WT and KO), p < 0.05, two-way ANOVA.

Since STING induces an inflammatory profile in macrophages and *B*. *abortus* induces HIF-1α expression and stabilization in a STING-dependent manner, we further investigated the linkage between HIF-1α and macrophage metabolic profile. First, we demonstrated that *B*. *abortus* induces HIF-1α stabilization in HIF-1α WT cells, which was not observed in HIF-1α KO control macrophages (Fig 2A). Moreover, HIF-1α KO macrophages showed reduced expression of the inflammatory markers CCR7 and NOS2, and enhanced expression of the anti-inflammatory marker YM1, albeit no difference was observed regarding ARG1 expression (Fig 2B). In addition, HIF-1α KO mice infected with *B*. *abortus* exhibited reduced frequency of splenic CD80^+^ and NOS2^+^ inflammatory macrophages in comparison with HIF-1α WT during the early phase of the infection (Fig 2C). Moreover, the frequency of NOS2^+^ in HIF-1α KO macrophages was also diminished at the later phase of infection. However, no difference was observed between these experimental groups regarding the frequency of anti-inflammatory macrophages in the spleen at 2 and 4 weeks post-infection (Fig 2C). Taken together, these results indicate that HIF-1α contributes to the induction of M1-like macrophages during the early phase of the infection. Gating strategy is shown in S2 Fig and representative 2D-plots of each analysis are shown in S4 Fig.

It was earlier reported that macrophage metabolic profile is decisive for the outcome of bacterial infections [19,20]. Therefore, to gain insight into the role of HIF-1α in host defense against *B*. *abortus*, HIF-1α WT and HIF-1α KO infected mice were assessed for bacterial load in the spleen. Indeed, HIF-1α KO mice showed enhanced bacterial load at 2 weeks post-infection and no difference at a later infection stage (Fig 2D). This data indicates that HIF-1α participates in the control of the infection during its initial phase, when this transcriptional factor augments the frequency of both CD80^+^ and NOS2^+^ markers of inflammatory macrophages in spleens (Fig 2C).

Moreover, a hallmark of the inflammatory phenotype of macrophages is their switch from ATP production by OXPHOS to glycolytic metabolism, a process that is regulated by HIF-1α, among other factors [6,32,33]. Thus, we investigated HIF-1α participation in macrophage switch into glycolysis durin*g B*. *abortus* infection. Initially, we assessed the expression of *GLUT1* and demonstrated that HIF-1α KO infected macrophages showed reduced expression of this glucose transporter (Fig 2E), suggesting that HIF-1α might participate in the induction of a glycolytic phenotype in macrophages infected with *B*. *abortus*. To further explore these data, we evaluated the metabolic feature of macrophages using a metabolic glycolytic rate assay (Seahorse extracellular flux analyzer). In this assay, the total proton efflux rate (PER) correlates to glycolysis, measured by the extracellular acidification rate (ECAR). The combination of protons together with lactate leads to medium acidification. The blockage of mitochondrial respiration achieved by the addition of rotenone and antimycin A results in obstruction of the TCA cycle, which allows the determination of the acidification rate that is derived from the CO_2_ produced exclusively by the mitochondria, termed mitoPER [34]. Hence, the specific glycolytic acidification is revealed by calculating the glycolytic proton efflux rate (termed glycoPER), which is the subtraction of the contribution of mitoPER from the total PER [34]. The inhibitor 2-DG is added to confirm that acidification is provided by the glycolytic pathway, since 2-DG blocks the glucose flux. In that context, macrophages derived from HIF-1α WT and HIF-1α KO mice were infected or not with *B*. *abortus*, and the total PER was determined and is shown in S5A Fig. The glycoPER was calculated as described above, excluding acidification contribution from mitochondrial respiration. Together, total PER and glycoPER revealed that *B*. *abortus*-infected macrophages displayed a higher proton flux rate, i.e. increased extracellular medium acidification (Figs S5A and 2F). Furthermore, addition of the inhibitor 2-DG blocked proton flux rate. In addition, from time-course measurements, we quantified the basal and compensatory glycolysis of macrophages. The basal term refers to glycolysis levels observed before OXPHOS blockage by rotenone and antimycin A. The compensatory glycolysis is achieved after OXPHOS inhibition, as it forces the cell to use glycolysis to accomplish ATP demand whereas mitochondrial ATP synthase is no longer producing this energy-carrying molecule. Our results revealed that infection with *B*. *abortus* boosts the basal and compensatory glycolysis in HIF-1α WT and HIF-1α KO macrophages (Fig 2G). Notably, this phenomenon is reduced in HIF-1α KO infected macrophages in comparison with WT cells (Fig 2G). Additionally, the respiratory profile of HIF-1α WT and HIF-1α KO macrophages infected or not with *B*. *abortus* was determined through oxygen consumption rate (OCR) quantification in that same time course experiment (S5B Fig). Thus, we quantified the basal respiration, which is in this case the OCR measured before the addition of any mitochondrial respiratory inhibitors minus the non-mitochondrial respiration (minimum rate measurement after the addition of these inhibitors). HIF-1α WT derived macrophage infected displayed a lower basal respiration in comparison with WT non-infected and HIF-1α KO cells (S5C Fig). This lower basal respiration observed in HIF-1α WT infected macrophages reflects the reduced mitoPER value in comparison with other groups (S5D Fig), indicating that HIF-1α signaling diminishes OXPHOS upon *B*. *abortus* infection and consequently mitochondrial-produced CO_2_. Together, these results indicate that the HIF-1α pathway influences the inflammatory/glycolytic profile that occurs in macrophages upon infection with *B*. *abortus*.

### HIF-1α augments inflammasome activation and NO production in *Brucella*-infected macrophages

**Fig 3:**
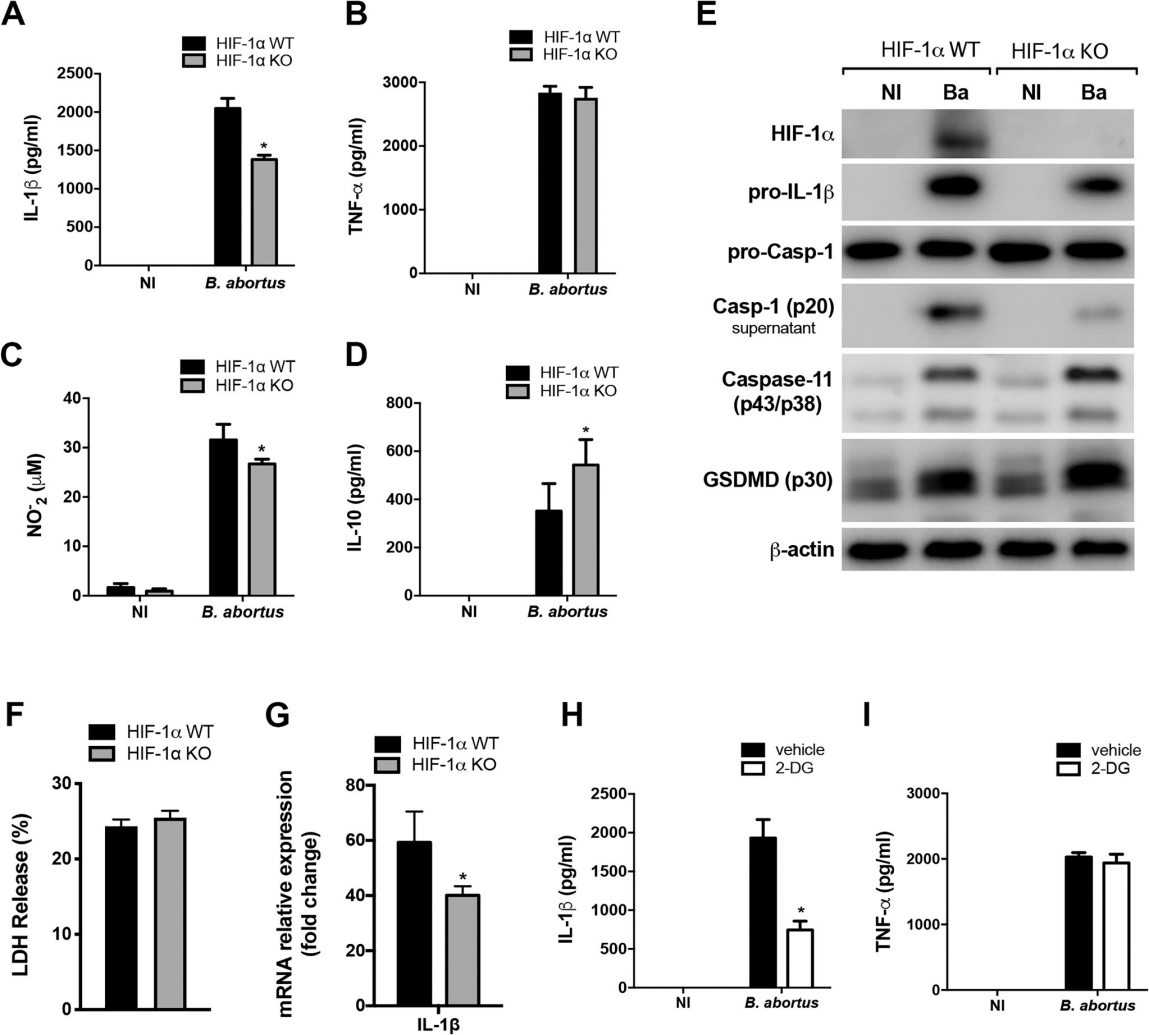
HIF-1α augments IL-1β release and NO production in response to *B*. *abortus* infection. IL-1β **(A)**, TNF-α **(B)** and IL-10 **(D)** produced by macrophages derived from HIF-1α WT and HIF-1α KO mice, non-infected (NI) or infected with *B*. *abortus*, detected in cell supernatants using ELISA. **(C)** NO_2_^−^ (nitrite) accumulation in the media of macrophages derived from HIF-1α WT and HIF-1α KO mice, non-infected (NI) or infected with *B*. *abortus*, measured by Griess reaction. **(E)** Western blot analysis of HIF-1α, pro-IL-1β, pro-caspase-1, caspase-11 and p30 fragment of GSDMD in cell lysates and caspase-1 (p20 subunit) in supernatants from macrophages derived from HIF-1α WT and HIF-1α KO mice, non-infected (NI) or infected with *B*. *abortus* (Ba). Equal loading was controlled by measuring β-actin in the corresponding cell lysates. **(F)** Cell death was determined by measuring the percentage of LDH release by *B*. *abortus*-infected macrophages derived from HIF-1α WT and HIF-1α KO mice. **(G)** IL-1β expression levels determined by real-time RT-PCR in *B*. *abortus*-infected macrophages derived from HIF-1α WT and HIF-1α KO mice. IL-1β released **(H)** and TNF-α secreted **(I)** from macrophages derived from C57BL/6 mice, non-infected (NI) or infected with *B*. *abortus*, and non-treated (vehicle) or pretreated with 1 mM 2-DG, detected in cell supernatants using ELISA. The data (A-I) are representative of three independent experiments. The data (A-D and H-I) are presented as mean ± SD, *p < 0.05, two-way ANOVA. The data (F-G) are presented as mean ± SD, *p < 0.05, Student’s t test.

An emerging body of evidence revealed that HIF-1α and HIF-1α-induced glycolysis act as critical pathways in modulating immune responses [35,36]. For instance, HIF-1α drives the expression of inflammatory genes during macrophage activation [6]. Therefore, to gain further insight into the role of HIF-1α in sustaining the inflammatory profile of macrophages and in macrophage response against *B*. *abortus* infection, we evaluated the production of inflammatory cytokines. Our results showed that the absence of HIF-1α reduced IL-1β release (Fig 3A), whereas TNF-α secretion was unaffected (Fig 3B). Additionally, since NO is another classical marker of the M1-like inflammatory macrophages profile [37], we also evaluated NO production in infected cells. The results showed that HIF-1α KO infected macrophages displayed a decreased NO production in comparison with HIF-1α WT infected cells (Fig 3C). In contrast, HIF-1α limited the production of the anti-inflammatory cytokine IL-10 (Fig 3D), which reinforces the involvement of HIF-1α in sustaining an inflammatory phenotype upon *B*. *abortus* infection.

In addition, HIF-1α KO infected macrophages displayed reduced pro-IL-1β (Fig 3E) which is consistent with reduced *IL-1β* gene expression (Fig 3G). Besides, the intracellular protein level of pro-caspase-1 remained unaltered while activated caspase-1 (p20 subunit in supernatant) protein levels was reduced in HIF-1α KO infected macrophages (Fig 3E), indicating reduced inflammasome assembly. Taken together, these results suggest that both signal 1 and 2 are disrupted in inflammasome activation in HIF-1α KO macrophages upon *B*. *abortus* infection. Moreover, our group previously described that IL-1β release by macrophages infected with *B*. *abortus* is partially dependent on activation of caspase-11 and gasdermin D (GSDMD) [38]. This non-canonical inflammasome activation leads to pyroptosis and lactate dehydrogenase (LDH) release [38]. Evaluation of the role of HIF-1α in this process shows that the intracellular protein level of caspase-11, GSDMD cleavage (generation of the p30 fragment) (Fig 3E) and LDH release (Fig 3F) were unaffected by the absence of HIF-1α. Thus, HIF-1α is dispensable for non-canonical inflammasome activation in *B*. *abortus*-infected macrophages.

Furthermore, inhibition of glucose flux in macrophages using 2-DG also diminished IL-1β release promoted by *B*. *abortus* (Fig 3H), whereas TNF-α secretion was unaffected (Fig 3I). These results indicate that while TNF-α secretion remains unaltered, the inhibition of either HIF-1α or glucose flux reduces IL-1β release in infected macrophages, demonstrating the relevant role of the HIF-1α-glycolysis axis in the activation of the inflammasome, a common inflammatory response during *B*. *abortus* infection [39].

### Limiting HIF-1α degradation shifts macrophage profile during *B*. *abortus* infection

**Fig 4:**
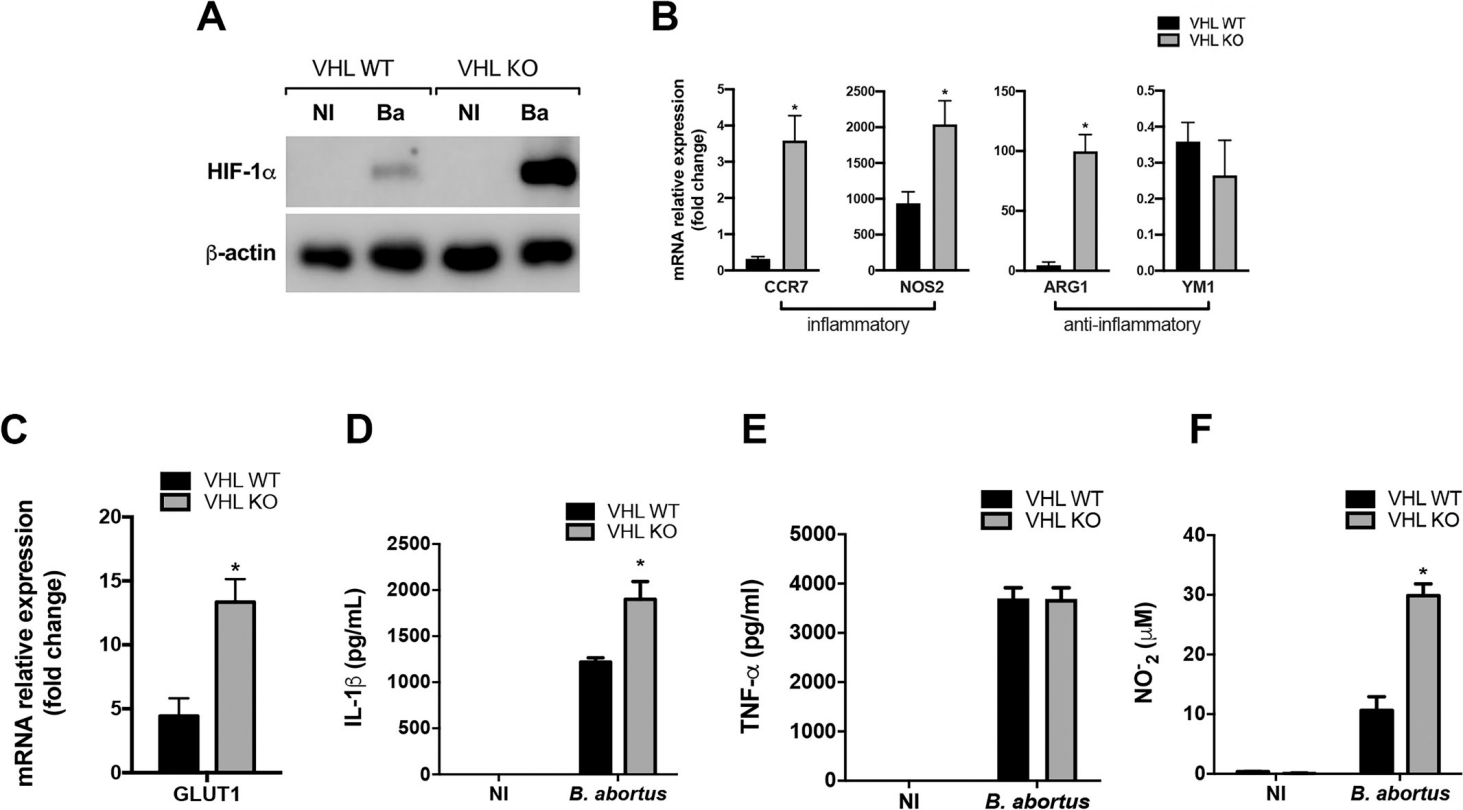
Limiting HIF-1α degradation induces an inflammatory profile in infected macrophages. **(A)** Western blot analysis of HIF-1α in cell lysates from macrophages derived from VHL WT and VHL KO mice, non-infected (NI) or infected with *B*. *abortus* (Ba). Equal loading was controlled by measuring β-actin in the corresponding cell lysates. **(B)** CCR7, NOS2, ARG1, and YM1 expression levels determined by real-time RT-PCR in macrophages derived from VHL WT and VHL KO mice infected with *B*. *abortus*. **(C)** GLUT1 expression levels determined by real-time RT-PCR in macrophages derived from VHL WT and VHL KO mice infected with *B*. *abortus*. IL-1β released **(D)** and TNF-α secreted **(E)** from macrophages derived from VHL WT and VHL KO mice, non-infected (NI) or infected with *B*. *abortus*, detected in cell supernatants using ELISA. **(F)** NO_2_^−^ (nitrite) accumulation in the media of macrophages derived from VHL WT and VHL KO mice, non-infected (NI) or infected with *B*. *abortus*, measured by Griess reaction. The data (A-F) are representative of three independent experiments. The data (B and C) are presented as mean ± SD, *p < 0.05, Student’s t test. The data (D-F) are presented as mean ± SD, *p < 0.05, two-way ANOVA.

The von-Hippel Lindau (VHL) tumor suppressor protein is a component of an E3 ubiquitin ligase that functions as a master regulator of HIF-1α activity by targeting the hydroxylated HIF-1α subunit for proteasomal degradation [40,41]. To confirm the role of HIF-1α in inducing an inflammatory profile in macrophages, we used mice with conditional deletion in myeloid cells (VHL KO), whose VHL deletion limits HIF-1α degradation. VHL KO macrophages showed enhanced HIF-1α protein levels during *B*. *abortus* infection when compared with VHL WT cells (Fig 4A). In contrast to what we observed for HIF-1α KO, VHL KO infected macrophages exhibited enhanced expression of the inflammatory markers CCR7 and NOS2 (Fig 4B) and augmented GLUT1 expression when compared with VHL WT cells (Fig 4C). Furthermore, VHL KO infected macrophages also displayed increased expression of the M2-related marker ARG1, albeit no difference was observed regarding the expression of YM1 (Fig 4B). Additionally, VHL KO infected macrophages displayed enhanced IL-1β release (Fig 4D) with no alteration in TNF-α levels (Fig 4E), and increased NO production (Fig 4F). Thus, the lack of VHL protein dictates higher HIF-1α protein levels and promotes a modified activation profile in macrophages. These findings reinforce the contribution of HIF-1α in reprogramming macrophage function towards an inflammatory profile upon *B*. *abortus* infection.

### STING increases the intracellular succinate concentration that leads to HIF-1α stabilization and IL-1β release

STING activation promoted by *B*. *abortus* infection triggers type I interferon (IFN) production in macrophages [25]. Thus, we evaluated the role of type I interferon response in the HIF-1α pathway using interferon-α/β receptor (IFNAR) KO mice. Notably, HIF-1α intracellular protein level (S6A Fig) and *HIF-1α* gene expression (S6B Fig) in macrophage were unaltered by IFNAR absence. In addition, treatment of STING KO mice with recombinant IFN-β (rIFN-β) failed to restore HIF*-1α* gene expression (S6C Fig), suggesting that type I interferon response is not involved in the HIF-1α pathway during *B*. *abortus* infection.

**Fig 5:**
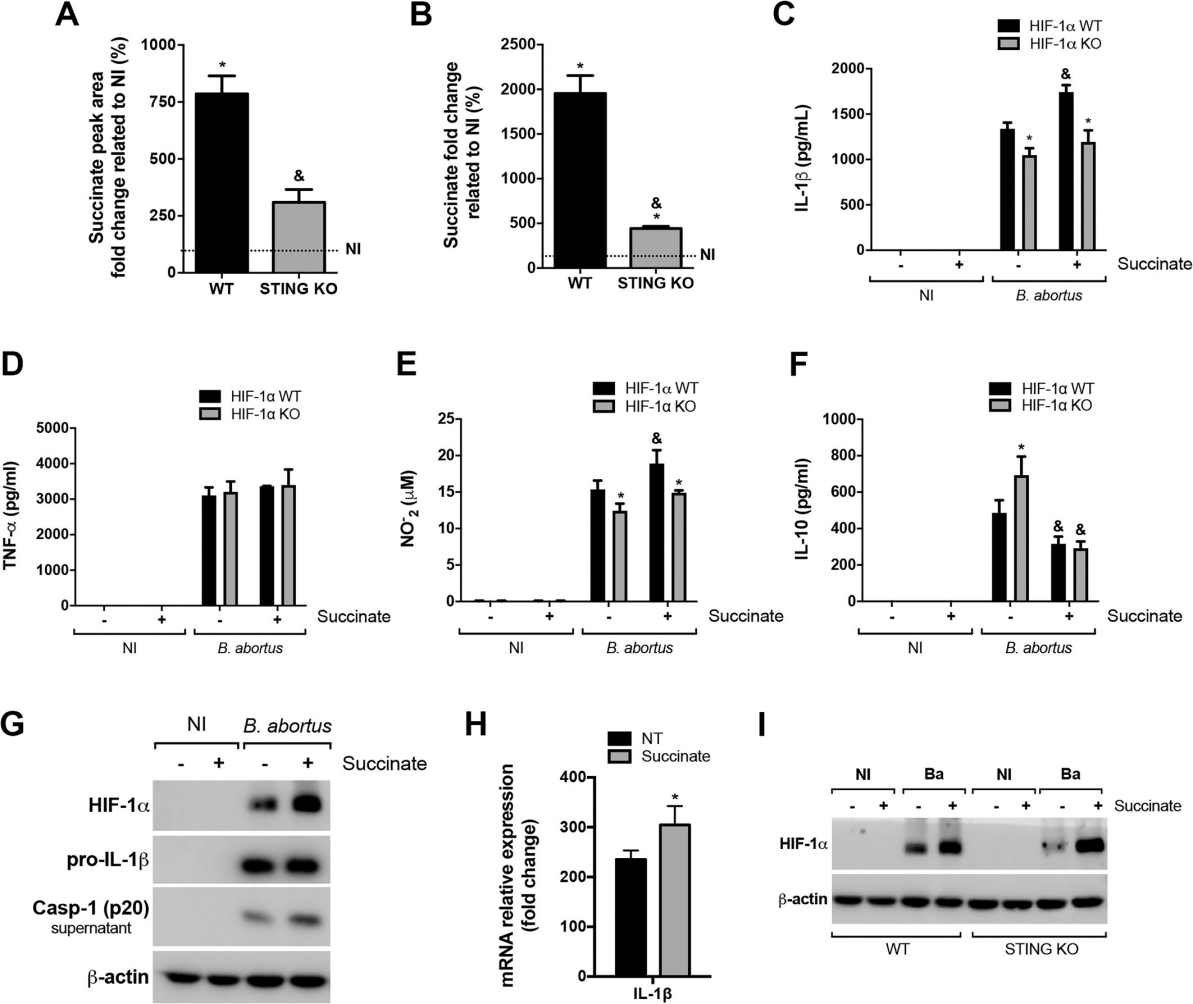
STING increases the intracellular succinate concentration that leads to HIF-1α stabilization and IL-1β release. Measurements of intracellular succinate levels by **(A)** liquid chromatography-mass spectrometry and by **(B)** Succinate Colorimetric Assay Kit in *B*. *abortus*-infected macrophages derived from C57BL/6 (WT) and STING KO mice. IL-1β **(C)**, TNF-α **(D)** and IL-10 **(F)** produced by macrophages derived from HIF-1α WT and HIF-1α KO mice, pretreated or not with succinate (5 mM) and then non-infected (NI) or infected with *B*. *abortus*, detected in cell supernatants using ELISA. **(E)** NO_2_^−^ (nitrite) accumulation in the media of macrophages derived from HIF-1α WT and HIF-1α KO mice, pretreated or not with succinate (5 mM) and then non-infected (NI) or infected with *B*. *abortus*, measured by Griess reaction. **(G)** Western blot analysis of HIF-1α and pro-IL-1β in cell lysates and caspase-1 (p20 subunit) in supernatants from macrophages derived from C57BL/6 (WT) mice, pretreated or not with succinate (5 mM) and then non-infected (NI) or infected with *B*. *abortus*. Equal loading was controlled by measuring β-actin in the corresponding cell lysates. **(H)** IL-1β expression levels determined by real-time RT-PCR in *B*. *abortus*-infected macrophages derived from C57BL/6 (WT) mice, non-treated (NT) or pretreated with succinate (5 mM). **(I)** Western blot analysis of HIF-1α in cell lysates from C57BL/6 (WT) and STING KO mice, pretreated or not with succinate (5 mM) and then non-infected (NI) or infected with *B*. *abortus* (Ba). Equal loading was controlled by measuring β-actin in the corresponding cell lysates. The data (A-I) are representative of three independent experiments. The data (A and B) are presented as mean ± SD, & (comparison between WT and KO) or * [comparison between non-infected (NI, set to 100%) and infected], p < 0.05, one-way ANOVA. The data (C-F) are presented as mean ± SD, * (comparison between WT and KO) or & (comparison between non-treated and succinate-treated), p < 0.05, two-way ANOVA. The data (H) is presented as mean ± SD, *p < 0.05, Student’s t test.

The shift to a glycolytic profile in inflammatory macrophages leads to the accumulation of the metabolite succinate which stabilizes the transcription factor HIF-1α [6–8]. Hence, we hypothesized that succinate may be involved as a signal to trigger a STING-dependent inflammatory state in macrophages. *B*. *abortus* infection led to succinate accumulation in WT macrophages that was reduced in STING KO infected cells (Fig 5A and 5B), confirming the contribution of STING in inducing an inflammatory profile in macrophages during infection. Considering that succinate accumulates during infection, we unraveled the effects of this metabolite in innate immune responses of *B*. *abortus*-infected macrophages. Pretreatment with succinate enhanced IL-1β release in infected-macrophages, a process that occurs, at least in part, in a HIF-1α-dependent manner (Fig 5C). However, succinate treatment did not alter TNF-α secretion (Fig 5D). In addition, succinate-enhanced NO production was also partially dependent on HIF-1α (Fig 5E). Further, succinate reduced the production of IL-10, but in that case, the mechanism seems to occur independently of HIF-1α since this phenomenon is observed in both WT and KO cells (Fig 5F). Importantly, succinate alone displayed no effect on cytokines or NO production by macrophages (Fig 5C–5F). Finally, to rule out the fact that succinate can enhance inflammation in immune cells via its specific receptor (GPR91) [12], we performed experiments using macrophages derived from GPR91 KO mice. Succinate treatment led to an enhanced release of IL-1β (S7A Fig) and NO production (S7C Fig) with no alteration in TNF-α levels (S7B Fig) in infected macrophages, independently of GPR91.

Since succinate treatment affected IL-1β production on a HIF-1α-dependent manner, we evaluated succinate participation in inducing HIF-1α stabilization in infected macrophages. Notably, succinate treatment boosted HIF-1α protein levels in *B*. *abortus* infected-macrophages (Fig 5G). In addition, succinate also enhanced *IL-1β* gene expression (Fig 5H), although pro-IL-1β protein levels were not affected despite succinate treatment in infected macrophages (Fig 5G). Further, succinate enhanced caspase-1 (p20 subunit) protein levels in macrophages infected with *B*. *abortus*, which implies the involvement of this metabolite in inflammasome activation (Fig 5G). To further determine the role of STING-derived succinate in HIF-1α stabilization, we treated STING KO macrophages with succinate and infected with *B*. *abortus*. Succinate treatment enhanced HIF-1α stabilization in infected STING KO macrophages (Fig 5I), supporting the link between HIF-1α and succinate accumulation promoted by STING.

All together, these data suggest that HIF-1α stabilization by increased succinate levels is one possible mechanism underlying the contribution of macrophage metabolic shift in inflammatory profile upon *B*. *abortus* infection.

### STING drives HIF-1α stabilization through mROS generation

**Fig 6:**
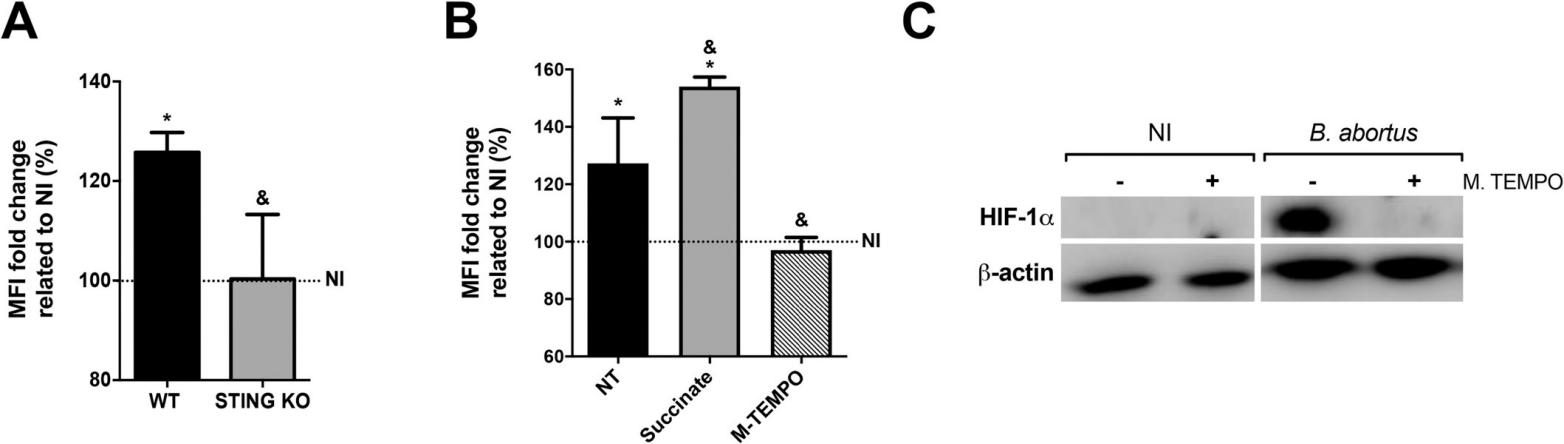
STING drives mROS production which augments HIF-1α stabilization. **(A)** MitoSOX Red flow cytometry analysis of mROS generation in *B*. *abortus*-infected macrophages derived from C57BL/6 (WT) and STING KO mice. **(B)** MitoSOX Red flow cytometry analysis of mROS generation in *B*. *abortus*-infected macrophages derived from C57BL/6 (WT) pretreated with succinate (5 mM), Mito-TEMPO (M.TEMPO, 0.5 mM) or vehicle (non-treated, NT). **(C)** Western blot analysis of HIF-1α in cell lysates from macrophages derived from C57BL/6 (WT) pretreated or not with Mito-TEMPO (M.TEMPO, 0.5 mM) and then non-infected (NI) or infected with *B*. *abortus*. Equal loading was controlled by measuring β-actin in the corresponding cell lysates. The data (A-C) are representative of three independent experiments. The data (A) is presented as mean ± SD, & (comparison between WT and KO) or * [comparison between non-infected (NI, set to 100%) and infected], p < 0.05, one-way ANOVA. The data (B) is presented as mean ± SD, & (comparison between NT and treated) or * [comparison between non-infected (NI, set to 100%) and infected], p < 0.05, one-way ANOVA.

Succinate oxidation by SDH favors mitochondrial mROS generation to support the inflammatory function of macrophages [11]. Therefore, to better understand how STING regulates the metabolic reprogramming of macrophages upon *B*. *abortus* infection, we evaluated mROS production in STING KO macrophages. Indeed, infection elevated mROS generation in WT macrophages while STING KO cells maintained the same level of mROS production as non-infected cells (Fig 6A). To further explore the mechanism by which STING regulates mROS generation in *Brucella*-infected macrophages, we evaluated the contribution of succinate to mROS production. Infection of cells pretreated with succinate promoted an increase in mROS generation when compared to non-treated cells (Fig 6B); while pretreatment with Mito-TEMPO, a scavenger specific for mROS, blocked mROS production by infected macrophages (Fig 6B). Additionally, it was recently shown that mROS can alter HIF-1α activity [11,42]. Hence, we addressed whether mROS could act as a signal to drive HIF-1α stabilization in infected macrophages. Indeed, *Brucella*-induced HIF-1α stabilization is reduced in infected macrophages pretreated with Mito-TEMPO (Fig 6C). Together, these results indicate that STING-induced mROS production acts as a stabilizing factor for HIF-1α in macrophages infected with *B*. *abortus*.

## Discussion

Host cells reprogram their metabolic pathways during the immune response against pathogenic infection, and these changes are directly linked to pathogen growth or restriction [43]. In that context, it was previously demonstrated that M2-like macrophages sustain increased levels of intracellular *Brucella* replication during chronic infection, a phenotype that relies on macrophage metabolic reprogramming induced by PPARγ activity, which causes an increase in intracellular glucose that supports intracellular bacteria growth [20]. Furthermore, we have recently shown that STING pathway is critical for host recognition and for triggering the innate immune response against *B*. *abortus* [25]. Here we demonstrated that STING activation contributes to shift macrophages towards an inflammatory profile through a mechanism associated with increased HIF-1α protein levels in *B*. *abortus* infected-macrophages.

It is well known that absence of HIF-1α results in profound impairment of myeloid cell function and bacterial killing [44,45]. Notably, HIF-1α regulates NO production and generation of inflammatory cytokines that drive pathogen clearance [27]. Our results indicate that HIF-1α drives inflammasome activation, NO production and enhances the frequency of CD80^+^ and NOS2^+^ macrophages during the initial phase of *B*. *abortus* infection which correlates with the control of splenic bacterial burden. Confirming the role of HIF-1α in sustaining the macrophage inflammatory phenotype, we also demonstrate that lack of VHL, which enhances HIF-1α protein levels, increases M1-like macrophage markers, IL-1β release and NO production. In accordance with our data showing the contribution of HIF-1α in the control of bacterial infection, it was likewise demonstrated that macrophage killing of the Gram-positive pathogen group A *Streptococcus* and the Gram-negative bacterium *Pseudomonas aeruginosa* was impaired upon HIF-1α deletion [46]. Additionally, loss of VHL in macrophages increased the intracellular killing of these pathogens. In this case, the bactericidal capacity of macrophages occurs through the regulation of NOS2 expression and NO production by HIF-1α and VHL [46]. Regarding *Brucella* infection, the involvement of NOS2 and NO radicals in inhibiting its intracellular replication in macrophages was likewise demonstrated [47]. Additionally, it was recently demonstrated that the multifunctional molecule thioredoxin-interacting protein (TXNIP) augments NOS2 expression and the production of NO which reduces *B*. *abortus* intracellular growth in macrophages [48]. Thus, our results indicate that the enhanced NOS2 expression, NO production and the inflammatory profile promoted by HIF-1α may support the control of *B*. *abortus* during the early phase of infection.

Glycolysis involves the conversion of glucose to pyruvate in the cytoplasm to generate only 2 ATP/molecule of glucose. This process is relatively inefficient when compared with the oxidation of acetyl-CoA to CO_2_ in the TCA cycle, which supply OXPHOS with high energy electron carrier molecules to produce a net of 36 ATP in total. In this scenario, the high glycolytic metabolism with a large consumption of glucose represents a metabolic strategy to meet the cell energy demands when the TCA cycle is disrupted [49]. Notably, we demonstrated that a main metabolic change evoked by HIF-1α activation during *B*. *abortus* infection is the increase in glycolysis and mitigation of OXPHOS, common features of M1-like macrophages [3], that were also previously correlated with HIF-1α activation [50]. Additionally, we observed here a higher PER and a diminished OCR in HIF-1α WT macrophages infected with *B*. *abortus* in comparison with HIF-1α KO cells, confirming that the increase in glucose utilization is dependent on HIF-1α. In this context, this robust glycolytic response prompted by HIF-1α may be interpreted as a key metabolic change essential for ATP generation to sustain the activation of immune cells during *B*. *abortus* response.

Regarding inflammasome activation, we show that lack of HIF-1α seems to interfere directly with pro-IL-1β synthesis and caspase-1 activation upon *B*. *abortus* infection, both required for a full mature IL-1β release. It was previously demonstrated that HIF-1α induces IL-1β expression in macrophages treated with LPS [6], corroborating the role of HIF-1α in activating the inflammasome. Furthermore, macrophages stimulated with LPS increase ATP production via glycolysis, decreasing the demand for ATP supplied by mitochondria, resulting in higher mitochondrial membrane potential and mROS production [11]. Previously, our group demonstrated that mROS is pivotal for NLRP3/caspase-1 inflammasome activation in macrophages during *B*. *abortus* infection [39]. Hence, the results here suggest that the increment in glycolysis promoted by HIF-1α upon *B*. *abortus* infection might sustain mROS production leading to caspase-1 activation. Accordingly, glycolysis inhibition by 2-DG decreases IL-1β release elicited by *B*. *abortus*-infected macrophages.

Succinate promotes HIF-1α stabilization by inhibiting PHD activity [51]. Moreover, succinate also induces mROS production following its oxidation by SDH [11], and mROS likewise inhibits the activity of PHD [52], leading to HIF-1α stabilization. In accordance, we demonstrated that STING induces mROS production which stabilizes HIF-1α in macrophages infected with *B*. *abortus*. Besides, STING increases intracellular succinate concentration upon *B*. *abortus* infection and succinate treatment augments mROS production. Thus, we hypothesize that STING induces mROS production, at least in part, by increasing the succinate concentration, which may enhance SDH activity. In addition, we also demonstrated that succinate treatment stabilizes HIF-1α which boosts NO production, IL-1β release and caspase-1 activation. Since we have previously shown that mROS is pivotal for caspase-1 activation [39], that effect may be correlated with succinate enhanced mROS production. Nevertheless, secretion of TNF-α is unaffected by HIF-1α or succinate treatment. In fact, it was previously shown that TNF-α is produced independently of succinate treatment in LPS-stimulated cells [11], corroborating the specificity of the response demonstrated here. Treatment of *B*. *abortus* infected-macrophages with succinate also increases the expression of IL-1β, although the level of pro-IL-1β protein was not affected. This apparent discrepancy may be a result of the increased caspase-1 activation observed in succinate treated-cells that possibly accounts for the relatively reduced intracellular levels of pro-IL-1β. Notably, GPR91 is dispensable for these effects promoted by succinate treatment. In addition, HIF-1α decreases the production of the anti-inflammatory cytokine IL-10 during *B*. *abortus* infection and succinate treatment aggravates this effect. However, it seems that additional mechanisms may participate in this case, since HIF-1α KO macrophages also show reduced IL-10 production in response to succinate treatment. For instance, the activity of SDH is required to limit IL-10 production in LPS-stimulated macrophages [11]. Thus, it seems that mROS production derived by succinate oxidation in the mitochondria limits IL-10 production by other mechanisms apart from HIF-1α stabilization.

In summary, the data presented here reveals that STING activation results in increase intracellular levels of succinate and mROS production which contributes to HIF-1α stabilization. HIF-1α augments GLUT1 expression, NO production, caspase-1 activation and IL-1β release, all hallmarks of inflammatory macrophages. In addition, HIF-1α increases glycolysis and reduces OXPHOS, important features for the induction of IL-1β release. The inflammatory macrophage profile and metabolic reprogramming orchestrated by STING via activation of the HIF-1α pathway may contribute to control *B*. *abortus* infection. Targeting these immunometabolic pathways may be an important resource for the development of future therapies to control inflammatory diseases such as *Brucella* infection.

## Materials and methods

### Ethics statement

All experiments involving animals were conducted in accordance with the Brazilian Federal Law number 11,794, which regulates the scientific use of animals in Brazil, the Institutional Animal Care and Use Committees (IACUC) guidelines, and the Animal Welfare Act and Regulations guidelines established by the American Veterinary Medical Association Panel on Euthanasia. Animals were fed, housed, and handled in strict agreement with these recommendations. All protocols were approved by the Committee for Ethics in Animal Experimentation (CEUA) at UFMG under permit #87/2017.

### Mice

Wild-type (WT) C57BL/6 mice were purchased from the Federal University of Minas Gerais (UFMG) and STING KO mice were described previously [22]. HIF-1α conditional knockout mice in their myeloid cell lineage, termed HIF-1α KO (LysM-Cre^+/-^/HIF-1α^fl/fl^); HIF-1α-non-deletable littermate controls negative for Cre recombinase, named HIF-1α WT (LysM-Cre^-/-^/HIF-1α^fl/fl^); and GPR91 KO mice were kindly provided by Dr. Jose Carlos Alves-Filho (Ribeirao Preto Medical School, University of Sao Paulo, Brazil). VHL KO (LysM-Cre^+/-^/VHL^fl/fl^) mice and littermates negative for Cre recombinase VHL WT (LysM-Cre^-/-^/VHL ^fl/fl^) were kindly provided by Dr. Pedro M.M. Moraes-Vieira (State University of Campinas, Brazil). IFNAR KO mice were kindly provided by Dr. Russell Vance (University of California at Berkeley, USA). The animals were maintained at UFMG and used at 6–8 weeks of age.

### Bacterial strains

The virulent *Brucella abortus* strain S2308 was obtained from our laboratory collection. The bacterium was grown in Brucella broth (BB) medium (BD Pharmingen, San Diego, CA) for 3 days at 37°C under constant agitation prior to use. The culture OD at 600 nm was measured in a spectrophotometer to determine the bacterial number in the solution.

### Generation of bone marrow-derived macrophages

Macrophages were derived from bone marrow of indicated mice in L929-conditioned medium (LCCM) using an adapted protocol [53]. Briefly, bone marrow cells were harvested from the femurs and tibias and differentiated with DMEM (Gibco/Thermo Fisher Scientific, Waltham, MA) containing 10% fetal bovine serum (FBS) (Life Technologies), 20% LCCM, 1% HEPES (Life Technologies) and 100 U/ml penicillin-streptomycin (Life Technologies), at 37°C in 5% CO_2_. At day 7 of culture, when cells had completely differentiated into macrophages, they were seeded in culture plates and cultivated at 37°C in 5% CO_2_ in DMEM supplemented with 10% FBS, 1% HEPES and 100 U/ml penicillin-streptomycin. After 24 hrs of seeding, macrophages were infected as described below.

### Macrophage treatment and infection with *Brucella abortus*

Where applicable, cells were pretreated with 100 U/mL of a mouse recombinant interferon beta (rIFN-β) (Millipore, Burlington, MA) for 24 hrs, 1 mM of 2-deoxy-D-glucose (2-DG) (Sigma-Aldrich, St. Louis, MO) for 4 hrs, with 5 mM succinic acid (succinate) (Sigma-Aldrich) for 6 hrs or with 0.5 mM of the mitochondrial superoxide scavenger Mito-TEMPO (Sigma-Aldrich) for 1 hr [39]. Cultured cells were then infected *in vitro* with *B*. *abortus* at the multiplicity of infection (MOI) of 100:1 in DMEM (5.5 mM glucose, 2 mM L-glutamine and no pyruvate) supplemented with 1% FBS for 24 hrs at 37°C in 5% CO_2_. Transient transfections of macrophages were carried out using FuGENEHD (Promega, Madison, USA), following the manufacturer’s instructions. Cells were transfected with 2’,3’- cGAMP (3 mg/mL) (InvivoGen) and then incubated for 24 hrs at 37°C in 5% CO_2_. Culture supernatants and cell lysates were harvested and stored at -80°C until use.

### Real-time RT-PCR

Macrophages were seeded at a density of 5x10^5^ cells per well in 24-well plates, treated as indicated above and total RNA isolated using TRIzol (Invitrogen, Carlsbad, CA), in accordance with the manufacturer’s instructions. Afterwards, total RNA was treated with DNase I (Invitrogen) to remove genomic DNA followed by reverse transcription of 1 μg of total RNA using Illustra Ready-To-Go RT-PCR Beads (GE Healthcare, Chicago, IL) according to the manufacturer’s instructions. Real-time RT-PCR was performed using SYBR Green PCR master mix (Applied Biosystems, Foster City, CA) on a QuantStudio3 real-time PCR instrument (Applied Biosystems), using the following cycling parameters: 60°C for 10 min, 95°C for 10 min, 40 cycles of 95°C for 15 sec, and 60°C for 1 min, and a dissociation stage of 95°C for 15 sec, 60°C for 1 min, 95°C for 15 sec, and 60°C for 15 sec. The appropriate primers were used to amplify a specific fragment corresponding to specific gene targets as described below: IL-1β forward: 5′-TGACCTGGGCTGTCCAGATG-3′; IL-1β reverse: 5′-CTGTCCATTGAGGTGGAGAG-3′; CCR7 forward: 5’-GGTGGTGGCTCTCCTTGTCATT-3’, CCR7 reverse: 5’- GCTTTAAAGTTCCGCACGTCCTT-3’; NOS2 forward: 5’- AGCACTTTGGGTGACCACCAGGA-3’, NOS2 reverse: 5’-AGCTAAGTATTAGAGCGGCGGCA-3’; ARG1 forward: 5’-TGACATCAACACTCCCCTGACAAC-3’, ARG1 reverse: 5’- GCCTTTTCTTCCTTCCCAGCAG-3’; YM1 forward: 5’-GGGCATACCTTTATCCTGAG-3’, YM1 reverse: 5’-CCACTGAAGTCATCCATGTC-3’; GLUT1 forward: 5’-GCTGTGCTTATGGGCTTCTC-3’, GLUT1 reverse: 5’-CACATACATGGGCACAAAGC-3’; HIF-1α forward: 5’-GGGTACAAGAAACCACCCAT-3’, HIF-1α reverse: 5’-GAGGCTGTGTCGACTGAGAA-3’, and β-actin forward: 5’- GGCTGTATTCCCCTCCATCG-3’, β-actin reverse: 5’-CCAGTTGGTAACAATGCCATGT-3’. All data were analyzed using the threshold cycle method and were calculated as relative expression after normalization to the β-actin gene. All fold changes are expressed normalized to the non-infected control. Real-time RT-PCR measurements were conducted in triplicate.

### Western blot analysis

Macrophages were seeded at a density of 5x10^5^ cells per well in 24-well plates and treated as indicated above. Then, culture supernatants were collected, and cells were lysed with M-PER Mammalian Protein Extraction Reagent (Thermo Fisher Scientific) supplemented with 1:100 protease inhibitor mixture (Sigma-Aldrich). Equal amounts of protein of cell lysates or equal volume of supernatants were loaded onto 12% or 15% SDS-polyacrylamide gels and then transferred to nitrocellulose membranes (Amersham Biosciences, Uppsala, Sweden) according to standard techniques. Membranes were blocked for 1 hr in Tris-buffered saline (TBS) with 0.1% Tween-20 containing 5% nonfat dry milk and incubated overnight with primary antibodies at 4°C. Primary antibodies used included a monoclonal against HIF-1α (clone D1S7W, Cell Signaling Technology, Danvers, MA), a monoclonal antibody against IL-1β (clone 3A6, Cell Signaling Technology), a monoclonal antibody against gasdermin D (clone GN20-13, Genentech, South San Francisco, CA), a monoclonal antibody against caspase-11 (clone Flamy-1, Adipogen, San Diego, CA), and a monoclonal antibody against the p20 subunit of caspase-1 (clone Casper-1, Adipogen), all at a 1:1000 dilution. Loading control blot was performed using a monoclonal antibody anti–β-actin (clone 13E5, Cell Signaling Technology) at a 1:5000 dilution. The membranes were washed three times for 5 min in TBS with 0.1% Tween 20 and incubated for 1 hr at 25°C with the appropriate HRP-conjugated secondary antibody at a 1:1000 dilution. Immunoreactive bands were visualized using Luminol chemi-luminescent HRP substrate (Millipore, Burlington, MA) and analyzed using ImageQuant TL Software (GE Healthcare).

### Cytokine measurements, LDH release determination and nitric oxide assay

Macrophages were seeded at a density of 5 × 10^5^ cells per well in 24-well plates and treated as indicated above. Then, cell supernatants were harvested for cytokine, LDH and NO measurements. The production of murine cytokines such as IL-1β, IL-10 and TNF-α was performed using ELISA kits (R&D systems, Minneapolis, MN), according to the manufacturer’s instructions. The lactate dehydrogenase (LDH) activity in supernatant and lysates was measured using a CytoTox96 LDH release kit (Promega, Madison, WI), according to the manufacturer’s instructions. The NO assay was performed measuring the concentration of nitrite (NO_2_^−^), a stable metabolite of NO, using the Griess reagent method as previously described [54].

### Determination of bacterial burden in *B*. *abortus*-infected mice

Five mice from each group were infected *i*.*p*. with 1 x 10^6^ colony formation units (CFU) of *B*. *abortus* in 0.1 ml of saline (NaCl 0.9%). After indicated time points, animals were euthanized and their spleens harvested. Subsequently, spleens were weighed independently before maceration in 10 ml saline. Following maceration, a portion of splenic extract was 10-fold serially diluted and plated in duplicate on Brucella broth agar. After 3 days of incubation at 37°C, the number of CFU was determined. Results were expressed as the mean log CFU/g spleen of each group as previously described [55]. Alternatively, a second portion of splenic extract was prepared for flow cytometric analysis.

### Flow cytometric analysis to evaluate macrophage subsets

Four or five mice from each group were infected and spleen cells were harvested as described previously. After organs maceration, cells were treated with ammonium-chloride-potassium buffer (0.15 M NH_4_Cl, 1.0 mM KHCO_3_, 0.1 mM Na_2_ EDTA, pH 7.2) to lyse red blood cells. After washing, cells were adjusted to 1x10^6^ cells per well in RPMI medium supplemented with 10% FBS, 100 U/ml penicillin-streptomycin, in a 96-well plate, and incubated with Brefeldin A (1 μg/well; Sigma-Aldrich) during 2 hrs at 37°C, 5% CO2. Subsequently, cells were incubated for 20 min with anti-mouse CD16/CD32 (BD Bioscience, Franklin Lakes, NJ, USA; 1:30) diluted in phosphate buffered saline/bovine serum albumin 0.25% (PBS/BSA) at 4°C to block Fc receptors. Cells were then washed and stained for surface markers with a mixture of the following antibodies: rat IgG2a anti-murine F4/80 conjugated to biotin (clone BM8, BD Bioscience, San Diego, CA; 1:200); rat IgG2b anti-murine CD11b conjugated to APC-Cy7 (clone M1/70, BD Bioscience; 1:200); hamster IgG2 anti-murine CD80 conjugated to FITC (clone 16-10A1, BD Bioscience; 1:200), rat IgG2a anti-murine CD163 conjugated to PE (clone TNKUPJ, Thermo Fisher Scientific; 1:200), and rat IgG2a anti-murine CD206 conjugated to APC (clone C068C2, BioLegend, London, UK, 1:200). Streptavidin PerCP-Cy5.5 (Thermo Fisher Scientific, 1:50) was added when necessary. For intracellular staining, cells were fixed and permeabilized using BD Cytofix/Cytoperm Kit (BD Biosciences), according to the manufacturer’s instructions, and rat IgG2a anti-murine NOS2 conjugated to PE-Cy7 (clone CXNFT, Invitrogen; 1:50) were used. The applicable isotype controls were used. Finally, Attune Acoustic Focusing Cytometer (Life Technologies) was used to collect 500,000 events and data were analyzed using FlowJo Software (Tree Star, Ashland, OR, USA). Gating strategy is shown in S2 Fig and representative 2D-plots of each analysis are shown in S3 and S4 Figs. The expression of macrophage markers was evaluated inside CD11b^+^F4/80^+^ subset population.

### Seahorse glycolytic rate analysis

The XF96 Extracellular Flux Analyzer (Agilent, Santa Clara, CA) was used to determine the glycolytic profile of cells. Oxygen consumption rate (OCR), extracellular acidification rate (ECAR) and proton efflux rate (PER) were determined using the Glycolytic Rate Assay Kit (Agilent), according to the protocols provided by the manufacturer. Briefly, macrophages were seeded at a density of 1 x 10^5^ cells per well in the manufacture’s 96-well plate and allowed to attach overnight. Cells were then infected or not with *B*. *abortus* at a MOI of 100:1 for 24 hrs in normal cell culture medium. Prior to the assay, cells were washed tree times and media was changed to Seahorse XF DMEM medium pH 7.4 (Agilent), supplemented to contain 25 mM glucose, 4 mM L-glutamine and 2 mM pyruvate. The plate was allowed to equilibrate for 1 hr in a CO_2_-free incubator at 37 °C before loading into the Seahorse analyzer. Seahorse XF96 cartridges were hydrated according to the manufacturer’s instructions. Three measurements of OCR and ECAR were performed before injection of mitochondrial inhibitors (rotenone and antimycin A) and then three additional measurements were achieved before injection of the inhibitor 2-DG. Experiments were performed with 4 replicates of each condition. The bioenergetic parameters mitoPER and glycoPER were calculated as described elsewhere [34].

### Succinate measurements

Macrophages were seeded at a density of 1 x 10^6^ cells per well in 6-well plates and infected or not with *B*. *abortus* at a MOI of 100:1 for 24 hrs. Cells were then harvested, centrifuged and pelleted for metabolite extraction using an adapted protocol previously described [56]. Briefly, metabolites from 1.2 x 10^7^ cells were extracted with sequential addition of ice-cold methanol, chloroform and water (1:1:1, 4 mL:4 mL:4 mL), vortexing samples for 2 min after each solvent addition. The mixture was then centrifuged at 14000 x g for 20 min at 4°C. Finally, supernatants (polar aqueous phase) were collected, dried on SpeedVac and kept at -80°C until use. Intracellular succinate concentrations were measured with a Succinate Colorimetric Assay Kit (Sigma-Aldrich) according to the manufacturer’s instructions. Alternatively, succinate levels were determined by liquid chromatography-mass spectrometry. The high performance liquid chromatography (HPLC) analysis of samples was carried out using the HPLC Prominence 20A system model (Shimadzu, Kyoto, Japan). Dried extracts were resuspended in H_2_O with 0.1% (v:v) trifluoroacetic acid (TFA) solution and then separated by reverse phase chromatography on a Kinetex HPLC Column (C18, 50 x 4.6 mm) (Phenomenex, CA, USA) using a linear gradient (solvent A: H_2_O with 0.1% (v:v) TFA; solvent B: acetonitrile with 0.1% (v:v) TFA) at a flow rate of 0.7 mL/min. Detection of the eluted metabolites was achieved spectrophotometrically at 216 nm. We performed an external succinate standard running as a separate chromatography under exactly the same conditions in order to estimate succinate retention time. To confirm the identity of succinate in eluted fractions (from samples and standard chromatography), we assessed the molecular weight by mass spectrometry [matrix assisted laser desorption ionization-time of flight (MALDI-TOF) (Autoflex, Bruker Daltonics, Germany)]. The chromatogram peak area of succinate (arbitrary units) from samples was calculated by HPLC software and used as an estimate of succinate concentration. The data are expressed as fold change; specifically, succinate concentration of infected cells was related to succinate concentration of non-infected cells for each experimental group.

### Measurement of mitochondrial reactive oxygen species

The production of mROS was detected by flow cytometry in cells using the MitoSOX Red (Invitrogen) staining, a fluorescent dye specific for the detection of O_2_^-^ in the mitochondria of live cells. Macrophages were adjusted to a density of 5 x 10^5^ cells diluted in 200 μL of phenol red-free DMEM and added to 2.0 mL microcentrifuge tubes. Cells were then treated with 100 μL of 5 mM succinate, 0.5 mM Mito-TEMPO or vehicle and incubated at 37°C in 5% CO_2_ for 30 min. Subsequently, macrophages were infected or not with 50 μL of *B*. *abortus* at a MOI of 100:1 for 1 hr. Cells were then incubated with 50 μL 1 μM MitoSOX for 20 min protected from light. All incubation steps were performed at 37°C in 5% CO_2_. Subsequently, cells were washed three times with PBS, resuspended in 200 μL of PBS per tube and transferred to a 96-well plate. Immediately, mROS production was evaluated by flow cytometry using Attune Acoustic Focusing equipment and the results were analyzed using FlowJo software. The median fluorescence intensity (MFI) of MitoSOX, which correlates with mROS production, was calculated. The data is expressed as MFI fold change; specifically, mROS production by infected cells was relativized to mROS production by non-infected cell for each experimental group.

### Gene array analysis

Microarray transcripts analysis of *Brucella*-infected macrophages derived from WT and STING KO mice was performed as described earlier [25] at the Center of Computational Science, University of Miami. Gene Expression Omnibus accession number is GSE96071 (https://www.ncbi.nlm.nih.gov/geo/query/acc.cgi?token=uvcjkweipvyzhyb&acc=GSE96071). The macrophage genes which showed significant regulation upon *B*. *abortus* infection (upregulated, > = 4-fold; downregulated, <0.25 fold) were analyzed for biological pathway enrichment in the gene ontology (GO; http://www.geneontology.org) and the Kyoto Encyclopedia of Genes and Genomes (KEGG; http://www.genome.jp/kegg/) databases using the R statistical environment with enrichR package (https://www.R-project.org/).

### Statistical analysis

Graphs and data analysis were performed using GraphPad Prism 5 (GraphPad Software, San Diego, CA), using two-way ANOVA, one-way ANOVA, or Student’s t test, as indicated. All quantitative data are expressed as mean ± standard deviation. A p value less than 0.05 (p<0.05) was considered statistically significant.

## Supporting information

S1 Fig STING ligand induces NOS2 but not ARG1 marker expression. NOS2 **(A)** and ARG1 **(B)** expression levels determined by real-time RT-PCR in macrophages derived from C57BL/6 (WT) and STING KO mice and transfected with 2’,3’- cGAMP (3 mg/mL). The data (A-B) are representative of two independent experiments and are presented as mean ± SD, *p < 0.05, Student’s t test.
(PDF)

S2 Fig Gating strategy for identifying inflammatory and anti-inflammatory macrophages by *ex vivo* flow cytometry. Spleen cells were gated for “total cells population” and then analyzed for expression of CD11b^+^F4/80^+^ (macrophages). Subsequently, this gate was used to identify the inflammatory (CD80^+^ and NOS2^+^) and anti-inflammatory (CD163^+^ and CD206^+^) macrophages subsets.
(PDF)

S3 Fig Representative 2D-plots for WT and STING spleen cells from infected mice. Representative plots of analysis shown in Fig 1D of spleen cells from infected C57BL/6 (WT) and STING KO mice, at 2 or 4 weeks post-infection (wpi). Cell populations are presented as mean ± SD.
(PDF)

S4 Fig Representative 2D-plots for HIF-1α WT and HIF-1α KO spleen cells from infected mice. Representative plots of analysis shown in Fig 2C of spleen cells from infected HIF-1α WT and HIF-1α KO mice, at 2 or 4 weeks post-infection (wpi). Cell populations are presented as mean ± SD.
(PDF)

S5 Fig Metabolic reprogramming in infected macrophages requires HIF-1α. **(A)** Time-course quantification of the total PER in macrophages derived from HIF-1α WT and HIF-1α KO mice, non-infected (NI) or infected with *B*. *abortus* (Ba). **(B)** Time-course quantification of the OCR in macrophages derived from HIF-1α WT and HIF-1α KO mice, non-infected (NI) or infected with *B*. *abortus* (Ba). **(C)** Quantification of basal respiration in macrophages derived from HIF-1α WT and HIF-1α KO mice, non-infected (NI) or infected with *B*. *abortus* (Ba). Basal respiration represents the minimum OCR value before the addition of any mitochondrial respiratory inhibitors minus the non-mitochondrial respiration. **(D)** Quantification of mitoPER in macrophages derived from HIF-1α WT and HIF-1α KO mice non-infected (NI) or infected with *B*. *abortus* (Ba). The data (A-D) are representative of two independent experiments. The data (C-D) are presented as mean ± SD, * (comparison between NI and Ba) or & (comparison between WT and KO), p < 0.05, one-way ANOVA.
(PDF)

S6 Fig Type I IFN response is not involved in HIF-1α stabilization. **(A)** Western blot analysis of HIF-1α in cell lysates from macrophages derived from C57BL/6 (WT) and IFNAR KO and then non-infected (NI) or infected with *B*. *abortus* (Ba). Equal loading was controlled by measuring β-actin in the corresponding cell lysates. **(B)** HIF-1α expression levels determined by real-time RT-PCR in *B*. *abortus*-infected macrophages derived from C57BL/6 (WT) and IFNAR KO mice. **(C)** HIF-1α expression levels determined by real-time RT-PCR in *B*. *abortus* (Ba)-infected macrophages derived from C57BL/6 (WT) and STING KO mice, non-treated or pretreated with recombinant IFN-β (rIFNβ). The data (A-C) are representative of two independent experiments. The data (B) is presented as mean ± SD. The data (C) is presented as mean ± SD, & (comparison between non-treated and treated) or * (comparison between WT and KO), p < 0.05, two-way ANOVA.
(PDF)

S7 Fig Succinate drives IL-1β and NO production independently of GPR91. IL-1β **(A)** and TNF-α **(B)** produced by macrophages derived from C57BL/6 (WT) or GPR91 KO mice, pretreated or not with succinate (5 mM) and then non-infected (NI) or infected with *B*. *abortus*, detected in cell supernatants using ELISA. **(C)** NO_2_^−^ (nitrite) accumulation in the media of macrophages derived from C57BL/6 (WT) or GPR91 KO mice, pretreated or not with succinate (5 mM) and then non-infected (NI) or infected with *B*. *abortus*, measured by Griess reaction. The data (A-C) are representative of three independent experiments. The data (A-C) are presented as mean ± SD, & (comparison between non-treated and succinate-treated), p < 0.05, two-way ANOVA.
(PDF)
