# Supplementary figures and images for: STING regulates metabolic reprogramming in macrophages via HIF-1α during Brucella infection
Source: PLoS Pathog. 2021 May 14;17(5):e1009597. doi: 10.1371/journal.ppat.1009597 (PMC8153530; doi:10.1371/journal.ppat.1009597)

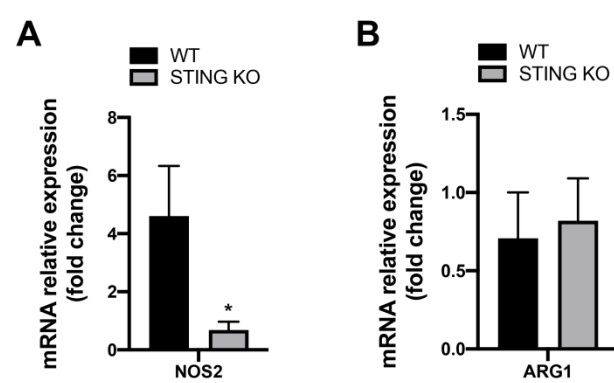

S1 Fig. STING ligand induces NOS2 but not ARG1 marker expression.

Supplement: S1 Fig — NOS2 (A) and ARG1 (B) expression levels determined by real-time RT-PCR in macrophages derived from C57BL/6 (WT) and STING KO mice and transfected with 2’,3’- cGAMP (3 mg/mL). The data (A-B) are representative of two independent experiments and are presented as mean ± SD, *p < 0.05, Student’s t test. (PDF) [file ppat.1009597.s001.pdf]

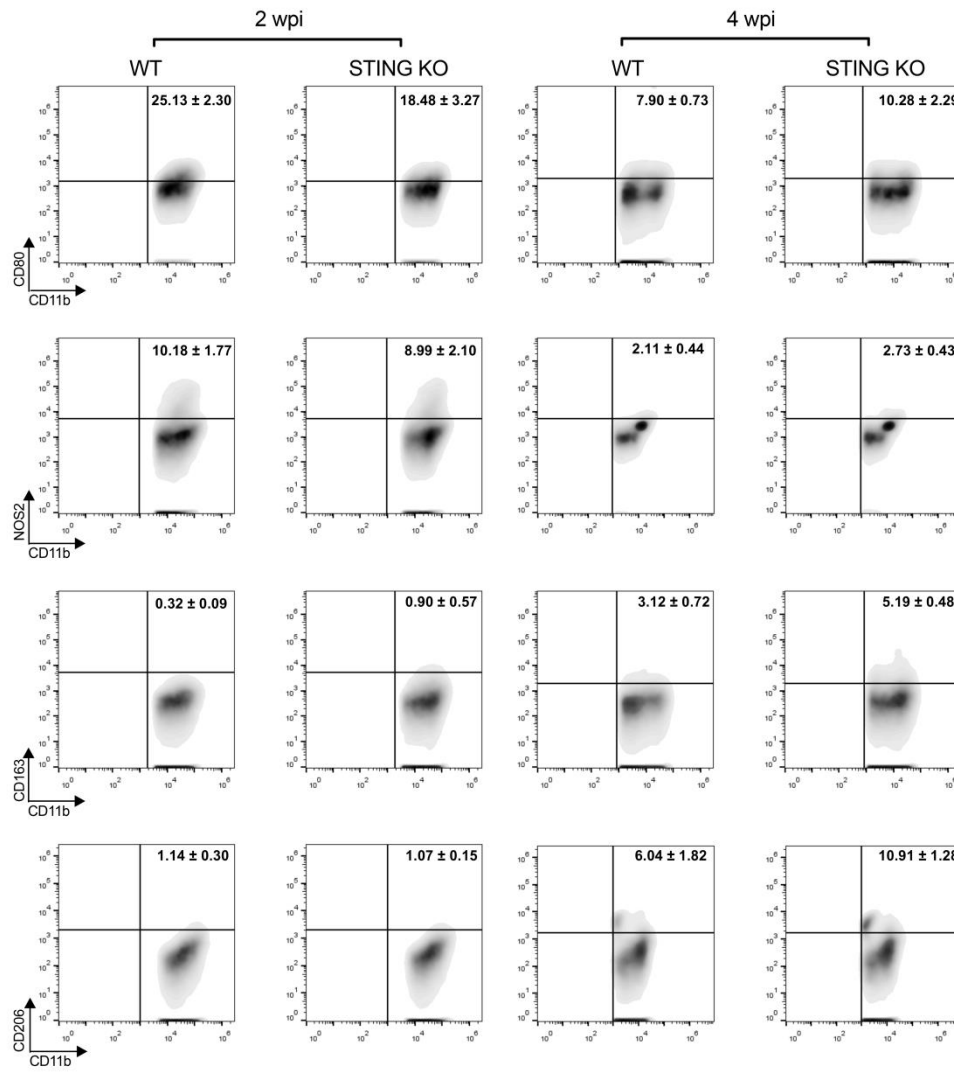

S3 Fig. Representative 2D-plots for WT and STING spleen cells from infected mice.

Supplement: S3 Fig — Representative plots of analysis shown in Fig 1D of spleen cells from infected C57BL/6 (WT) and STING KO mice, at 2 or 4 weeks post-infection (wpi). Cell populations are presented as mean ± SD. (PDF) [file ppat.1009597.s003.pdf]

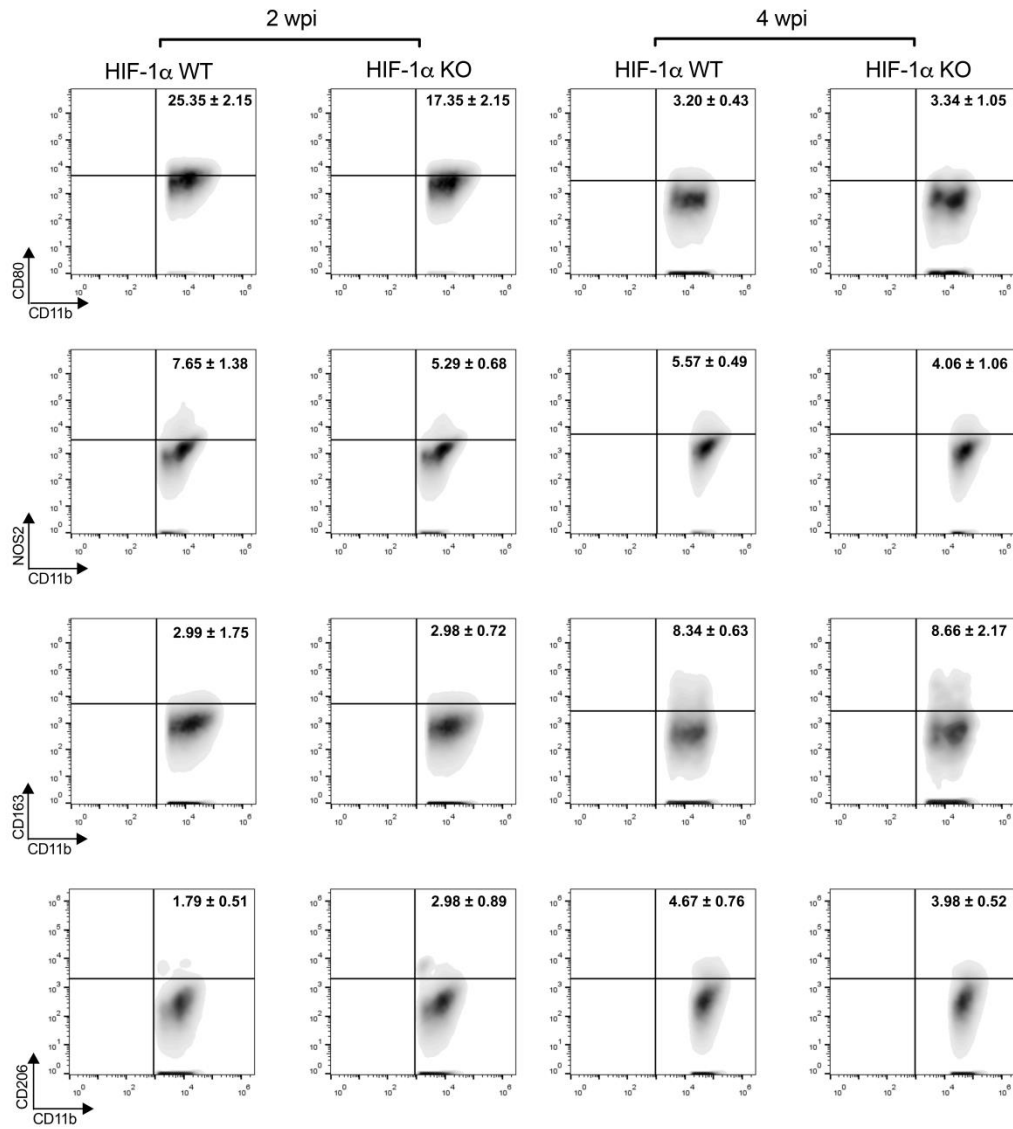

S4 Fig. Representative 2D-plots for HIF-1 $\alpha$  WT and HIF-1 $\alpha$  KO spleen cells from infected mice.

Supplement: S4 Fig — Representative plots of analysis shown in Fig 2C of spleen cells from infected HIF-1α WT and HIF-1α KO mice, at 2 or 4 weeks post-infection (wpi). Cell populations are presented as mean ± SD. (PDF) [file ppat.1009597.s004.pdf]

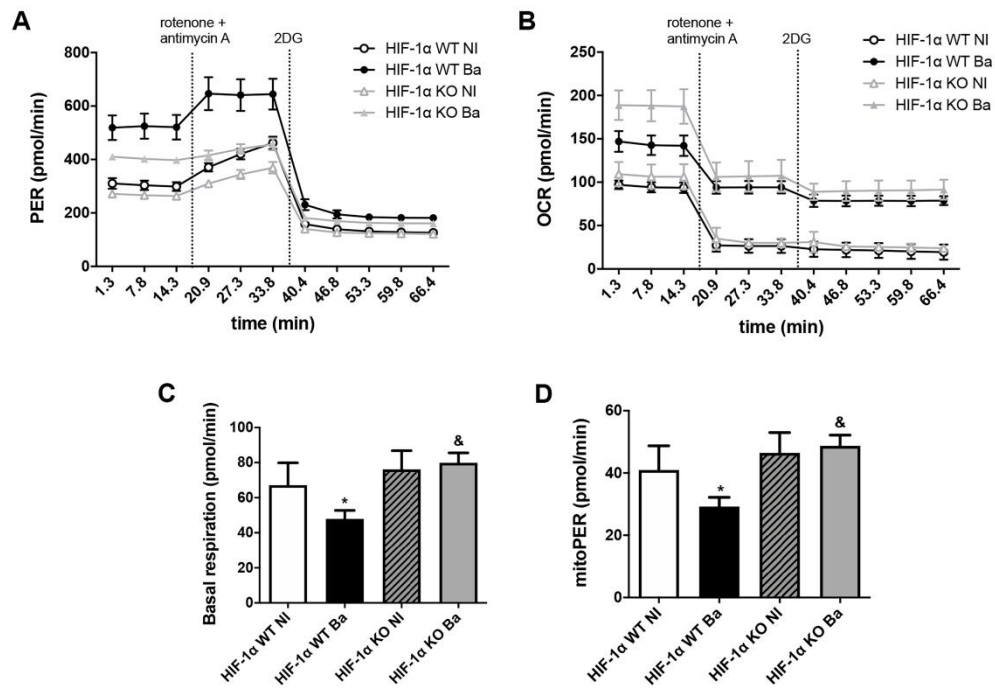

S5 Fig. Metabolic reprogramming in infected macrophages requires HIF-1 $\alpha$ .

Supplement: S5 Fig — (A) Time-course quantification of the total PER in macrophages derived from HIF-1α WT and HIF-1α KO mice, non-infected (NI) or infected with B. abortus (Ba). (B) Time-course quantification of the OCR in macrophages derived from HIF-1α WT and HIF-1α KO mice, non-infected (NI) or infected with B. abortus (Ba). (C) Quantification of basal respiration in macrophages derived from HIF-1α WT and HIF-1α KO mice, non-infected (NI) or infected with B. abortus (Ba). Basal respiration represents the minimum OCR value before the addition of any mitochondrial respiratory inhibitors minus the non-mitochondrial respiration. (D) Quantification of mitoPER in macrophages derived from HIF-1α WT and HIF-1α KO mice non-infected (NI) or infected with B. abortus (Ba). The data (A-D) are representative of two independent experiments. The data (C-D) are presented as mean ± SD, * (comparison between NI and Ba) or & (comparison between WT and KO), p < 0.05, one-way ANOVA. (PDF) [file ppat.1009597.s005.pdf]

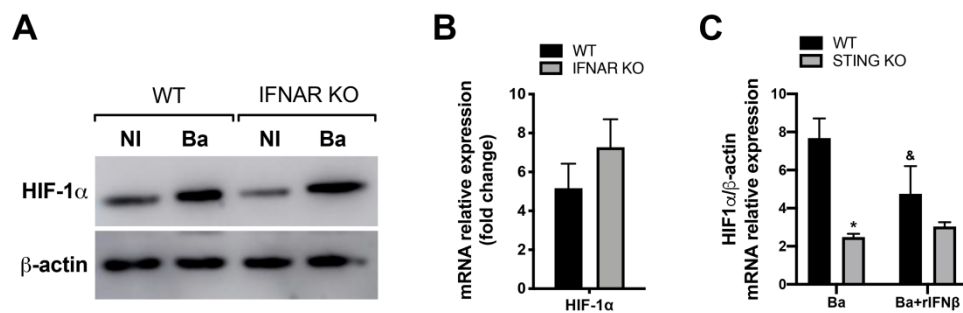

S6 Fig. Type I IFN response is not involved in HIF-1 $\alpha$  stabilization.

Supplement: S6 Fig — (A) Western blot analysis of HIF-1α in cell lysates from macrophages derived from C57BL/6 (WT) and IFNAR KO and then non-infected (NI) or infected with B. abortus (Ba). Equal loading was controlled by measuring β-actin in the corresponding cell lysates. (B) HIF-1α expression levels determined by real-time RT-PCR in B. abortus-infected macrophages derived from C57BL/6 (WT) and IFNAR KO mice. (C) HIF-1α expression levels determined by real-time RT-PCR in B. abortus (Ba)-infected macrophages derived from C57BL/6 (WT) and STING KO mice, non-treated or pretreated with recombinant IFN-β (rIFNβ). The data (A-C) are representative of two independent experiments. The data (B) is presented as mean ± SD. The data (C) is presented as mean ± SD, & (comparison between non-treated and treated) or * (comparison between WT and KO), p < 0.05, two-way ANOVA. (PDF) [file ppat.1009597.s006.pdf]

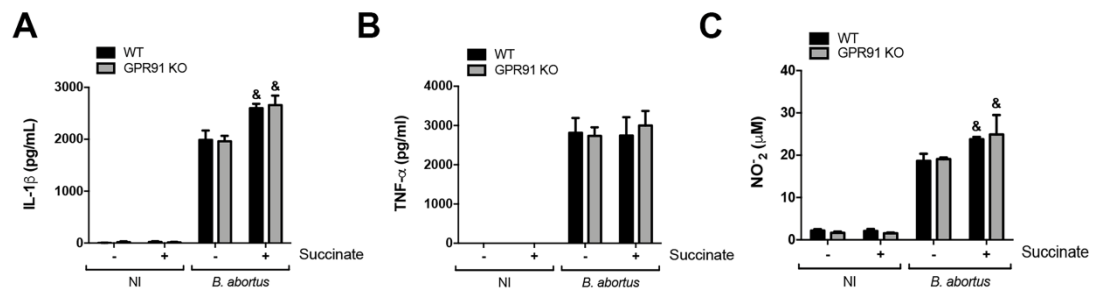

S7 Fig. Succinate drives IL-1 $\beta$  and NO production independently of GPR91.

Supplement: S7 Fig — IL-1β (A) and TNF-α (B) produced by macrophages derived from C57BL/6 (WT) or GPR91 KO mice, pretreated or not with succinate (5 mM) and then non-infected (NI) or infected with B. abortus, detected in cell supernatants using ELISA. (C) NO2− (nitrite) accumulation in the media of macrophages derived from C57BL/6 (WT) or GPR91 KO mice, pretreated or not with succinate (5 mM) and then non-infected (NI) or infected with B. abortus, measured by Griess reaction. The data (A-C) are representative of three independent experiments. The data (A-C) are presented as mean ± SD, & (comparison between non-treated and succinate-treated), p < 0.05, two-way ANOVA. (PDF) [file ppat.1009597.s007.pdf]
