# Supplementary material for: STING regulates metabolic reprogramming in macrophages via HIF-1α during Brucella infection
Source: PLoS Pathog. 2021 May 14;17(5):e1009597. doi: 10.1371/journal.ppat.1009597 (PMC8153530; doi:10.1371/journal.ppat.1009597)
Supplement: S2 Fig — Spleen cells were gated for “total cells population” and then analyzed for expression of CD11b+F4/80+ (macrophages). Subsequently, this gate was used to identify the inflammatory (CD80+ and NOS2+) and anti-inflammatory (CD163+ and CD206+) macrophages subsets. (PDF) [file ppat.1009597.s002.pdf]

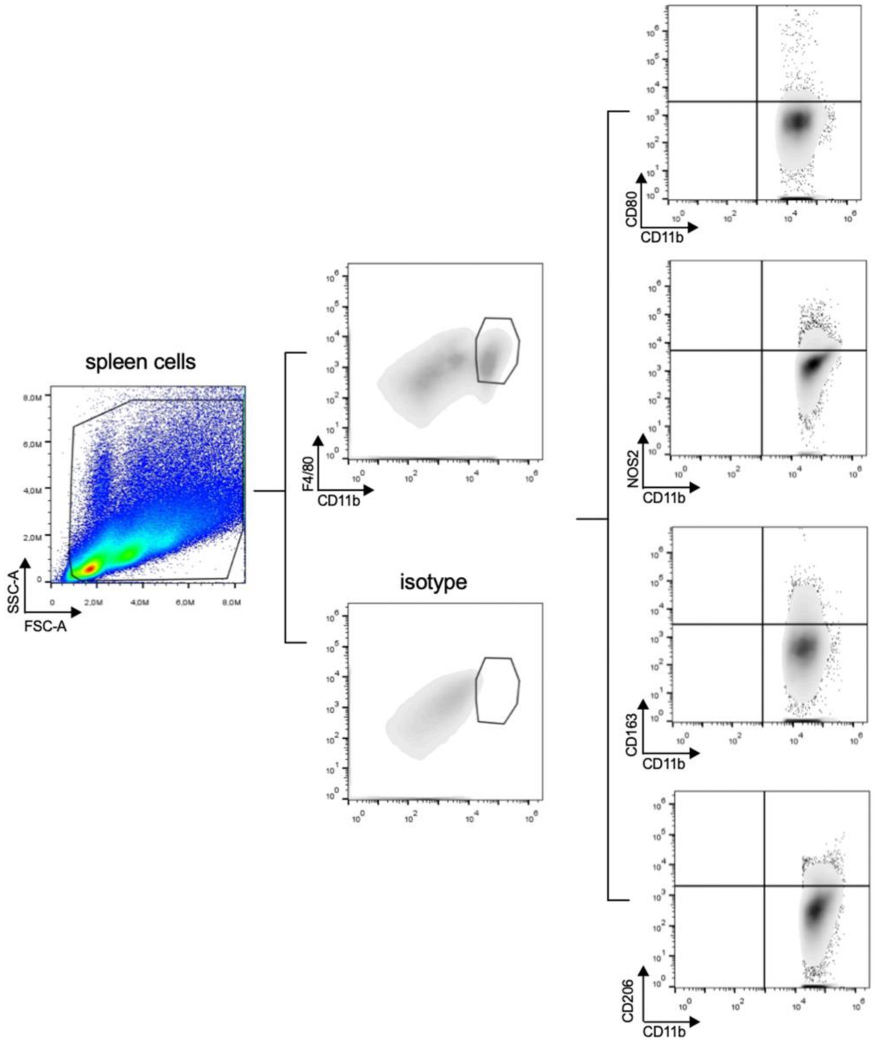

S2 Fig. Gating strategy for identifying inflammatory and anti-inflammatory macrophages by *ex vivo* flow cytometry.
